# Efficacy and safety of co-administered ivermectin plus albendazole for treating soil-transmitted helminths: A systematic review, meta-analysis and individual patient data analysis

**DOI:** 10.1371/journal.pntd.0006458

**Published:** 2018-04-27

**Authors:** Marta S. Palmeirim, Eveline Hürlimann, Stefanie Knopp, Benjamin Speich, Vicente Belizario, Serene A. Joseph, Michel Vaillant, Piero Olliaro, Jennifer Keiser

**Affiliations:** 1 Swiss Tropical and Public Health Institute, Basel, Switzerland; 2 University of Basel, Basel, Switzerland; 3 Basel Institute for Clinical Epidemiology and Biostatistics, Department of Clinical Research, University Hospital Basel, University of Basel, Basel, Switzerland; 4 Department of Parasitology, College of Public Health, University of the Philippines Manila, Metro Manila, Philippines; 5 World Health Organization, Geneva, Switzerland; 6 Luxembourg Institute of Health, Luxembourg, Luxembourg; 7 Special Programme for Research & Training in Tropical Diseases (TDR), World Health Organization, Geneva, Switzerland; Erasmus MC, NETHERLANDS

## Abstract

**Background:**

The soil-transmitted helminths (STH), *Ascaris lumbricoides*, *Trichuris trichiura* and hookworms, infect 1.5 billion people worldwide and cause an estimated burden of 3.3 million disability-adjusted life years (DALYs). Current control strategies focus on morbidity reduction through preventive chemotherapy (PC) but the most commonly used recommended drugs (albendazole and mebendazole) are particularly inefficacious against *T*. *trichiura*. This, together with the threat of emerging drug resistance, calls for new control strategies, including co-administration with other anthelminthics. Ivermectin plus albendazole is widely used against lymphatic filariasis, but its efficacy and safety against STH infections has not yet been fully understood.

**Methods and findings:**

We conducted a systematic literature review and meta-analysis on the efficacy and safety of ivermectin-albendazole co-administration in five different databases (i.e. PubMed, ISI Web of Science, ScienceDirect, CENTRAL and clinicaltrials.gov) from 1960 to January 2018. Four studies reporting efficacy of ivermectin-albendazole against STH infections and five studies on its safety met the selection criteria and were included for quantitative analysis. Ivermectin-albendazole was significantly associated with lower risk (risk ratio (RR) = 0.44, 95% confidence interval (CI) = 0.31–0.62) for *T*. *trichiura* infection after treatment compared to albendazole alone. The co-administration revealed no or only a marginal benefit on cure and egg reduction rates over albendazole alone for *A*. *lumbricoides* and hookworm infections. Adverse events (AEs) occurring after ivermectin-albendazole co-administration were mostly mild and transient. Overall, the number of individuals reporting any AE was not different (RR = 1.09, 95% CI = 0.87–1.36) in co-treated and albendazole-treated patients. However, although not statistically significant, sub-group analysis showed a tendency for slightly more AEs in patients with filariasis treated with ivermectin-albendazole compared to those treated with albendazole alone (RR = 1.29, 95% CI = 0.81–2.05).

**Conclusions:**

Our findings suggest a good tolerability and higher efficacy of ivermectin-albendazole against *T*. *trichiura* compared to the current standard single-dose albendazole treatment, which supports the use of this co-administration in PC programs. Large-scale definitive randomized controlled trials are required to confirm our results.

## Author summary

Millions of people worldwide are infected with intestinal worms known as soil-transmitted helminths (STHs). These include *Ascaris lumbricoides*, hookworm (*Ancylostoma duodenale* and *Necator americanus*) and *Trichuris trichiura*. Globally, the current main strategy to control these parasites is the periodical distribution of treatment to populations which are at particular risk of infection. However, the two drugs exclusively used in these mass treatment campaigns (albendazole and mebendazole) do not perform well against some of the STH species. Adding another drug, such as ivermectin, to albendazole holds promise for improved performance. This combination is already being widely used for the treatment of lymphatic filariasis. However, policy makers need evidence that this co-administration is efficacious and safe when taken together for STH infections. To fill this knowledge gap, we systematically reviewed the scientific literature for studies on the efficacy and safety of the co-administration of ivermectin and albendazole. We identified four studies reporting on the efficacy of the co-treated and five studies assessing their tolerability. Our results suggest that the co-administration performs significantly better than albendazole alone against *T*. *trichiura*–the STH species which is most difficult to treat–and is generally well-tolerated. Yet, we conclude that definitive evidence to support the use of ivermectin together with albendazole for STH will require more high-quality studies.

## Introduction

Soil-transmitted helminths (STHs) collectively cause the most widespread neglected tropical disease (NTD): nearly 1.5 billion people are infected with *Ascaris lumbricoides*, *Trichuris trichiura*, and/or hookworm (i.e. *Necator americanus* and *Ancylostoma duodenale*) in over 100 endemic countries [1, 2] and 3.3 million disability-adjusted life years (DALYs) are related to symptomatic infection, wasting, mild abdominopelvic problems and anemia [1, 3].

The World Health Organization (WHO) recommends large-scale, periodic distribution of safe and efficacious anthelminthic drugs as preventive chemotherapy (PC) to at-risk populations in endemic areas for morbidity control of STH infections [4, 5]. PC allows a reduction of infection intensities from heavy or moderate to light, hence preventing morbidity, rather than curing infection and/or interrupting transmission [5]. The groups at highest risk of STH infection-related morbidity are children, who are in a critical phase of growth and development, and women of childbearing age, including pregnant women, who have increased nutritional requirements during pregnancy and lactation [6].

Currently, STH infections are treated predominantly with the two benzimidazole drugs albendazole (400 mg) or mebendazole (500 mg) [7]. A meta-analysis published in 2017 showed that both drugs are highly efficacious against *A*. *lumbricoides* (cure rate (CR) = 96% with albendazole and CR = 96% with mebendazole), but less efficacious against hookworm (CR = 80% with albendazole and CR = 33% with mebendazole), and even less against *T*. *trichiura* (CR = 31% with albendazole and CR = 42% with mebendazole) [8]. Thus, it is crucial to increase efforts to explore alternative therapies to both increase efficacy for trichuriasis and delay the emergence of potential drug resistance in view of the massive drug pressure exerted by widespread use and dependence of these two drugs.

An additional anthelminthic drug, ivermectin, has been used widely in humans, either alone against onchocerciasis or in combination with albendazole against lymphatic filariasis (LF) since the late 1980s [7, 9]. This drug has played a key role in the elimination programs of these two NTDs [4]. In 2015 alone, more than 50 million school-aged children received ivermectin in addition to albendazole within the global program to eliminate LF [10]. It is not clear, however, how the LF program translates into clearing and/or reducing the intensity of STH infections [11]. There is indirect evidence of reduced STH burden in areas where albendazole and ivermectin have been co-administered [12]. While ivermectin alone is considered to have suboptimal efficacy against hookworm and *T*. *trichiura* infections [13–17] there are data indicating that the co-administration of ivermectin and albendazole can be more efficacious than single-drug regimens [18–20]. The co-administration of ivermectin and albendazole was therefore recently added to the WHO Essential Medicines List for the treatment of STH infection [21].

We conducted, for the first time, a systematic review and meta-analysis of the efficacy and safety of the co-administration of albendazole plus ivermectin compared to albendazole alone for treating STH infections. Post-treatment reactions are often related to disease triggered by parasite death [22] and it is thus important to know whether the tolerability is comparable in STH and LF infections. Furthermore, we analyzed efficacy measures based on individual patient data from three recent randomized controlled trials (RCTs). Our findings will help to inform improved treatment guidelines for STH infections.

## Methods

### Protocol and registration

The protocol of this systematic review, which is provided as a supplementary file (S1 File), was recorded and published in the International Prospective Register of Systematic Reviews (PROSPERO) online database, number CRD42017060710 (S2 File). The review and meta-analysis were conducted according to the Preferred Reporting Items for Systematic Reviews and Meta-Analysis (PRISMA) statement [23]. Reporting according to PRISMA guidelines are summarized in the checklist provided as a supplementary file (S3 File).

### Efficacy information sources and search strategy

A literature search without language restriction was performed in PubMed, ISI Web of Science and Science Direct (from 1960 to January 24, 2018) to identify clinical trials pertaining to the use of ivermectin in combination with albendazole for treating hookworm, *A*. *lumbricoides* and *T*. *trichiura*. The search terms included: “ivermect* [AND] albendaz* [AND] (hookworm [OR] trichuri* [OR] ascari* [OR] soil-transmitted helminth*) [AND] (cure* [OR] trial)”. Additionally, we performed a search using the keywords “ivermectin” and “albendazole” in the following databases and online repositories: Cochrane Central Register of Controlled Trials (CENTRAL) and ClinicalTrials.gov maintained by the National Library of Medicine (NLM) at the National Institutes of Health (NIH). The search strategy is detailed in supplementary file (S1 Text).

Individual patient data on efficacy of ivermectin combined with albendazole against STH infections from three published trials [18, 20, 24] were obtained through personal communication and were subjected to further in-depth analysis.

### Safety information sources and search strategy

To identify safety data from the co-administration of ivermectin and albendazole, a literature search was performed using databases from PubMed, ISI Web of Science and Science Direct (from 1960 to January 24, 2018) applying the following search terms: “ivermect* [AND] alben* [AND] combin* [AND] (adverse [OR] side effect* [OR] symptom*)”. No restrictions with regard to language, parasite species or study type were applied. Likewise to efficacy, we additionally searched the online databases and repositories of CENTRAL and ClinicalTrials.gov for documentations on safety using the keywords “ivermectin” and “albendazole” (S1 Text).

### Eligibility criteria

All retrieved references were screened by title and abstract for efficacy and safety information on ivermectin-albendazole co-administration in humans using the eligibility criteria detailed below. From the studies assessing efficacy, we selected RCTs, which tested the co-administration of ivermectin and albendazole against at least one STH (hookworm, *T*. *trichiura* and/or *A*. *lumbricoides*). We included only studies which administered the standard doses of the drugs (ivermectin: 200 μg/kg; albendazole: 400 mg) as recommended by the Essential Medicine List [21], and which assessed drug efficacy (follow-up survey) between 7 days and six weeks post-treatment. According to WHO, follow-up assessment of drug efficacy should take place between two and three weeks post-treatment because reinfection is common and long follow-up periods may prevent a clear distinction between poor efficacy and new infections [25]. However, to be more inclusive, we extended this period from 7 days to six weeks. The diagnostic method used in the studies was not part of the selection criteria.

The main eligibility criterion for potentially relevant studies on safety was reporting of any quantitative or qualitative data of adverse events (AEs) following administration of ivermectin in combination with albendazole in any clinical trial. Sample size varied among studies but was not considered as an inclusion/exclusion criterion. Case studies from medical reports were not considered due to non-representativeness of outcomes. Data published in reviews were included if they had not been identified in our literature search already. Additional relevant studies on safety identified through reviews or online repositories and not yet covered through the literature search were subsequently included. Due to the non-standardized approach to reporting safety information, we expanded the search to include different doses and different time points of AE assessment and follow-up (*vs*. efficacy for which there is a standardized approach recommended by WHO).

### Risk of bias within and across included studies

The quality and risk of bias of eligible efficacy studies was assessed at study level using the Cochrane risk of bias tool [26]. The assessment was based on six items included in the bias assessment tool: random sequence generation, allocation concealment (both define the selection bias), blinding of participants and personnel (performance bias), blinding of outcome assessment (detection bias), incomplete outcome data (attrition bias) and selective reporting (reporting bias). Each study was rated, for each of the items, as “high risk” or “low risk” of bias based on the criteria for judging risk of bias. If the study did not report sufficient detail to consider “high risk” or “low risk”, the risk of bias was classified as “unclear risk”.

For safety studies included in the meta-analysis the same Cochrane training tool for quality assessment was applied as detailed above. As there were fewer than 10 studies in each of the meta-analysis, the risk of bias across studies could not be assessed, as recommended by Sterne *et al*. (2011) [27].

### Data extraction

All references fulfilling the eligibility criteria were subjected to data extraction in duplicate by two independent reviewers (MSP and EH). For each study information on the publication (*i*.*e*., authors and year), general study-specific data such as type of study, country where the study took place, parasite species, participant data (*i*.*e*., age group, number of individuals), follow-up period and data collection method (*i*.*e*., repeated stool sampling for efficacy, passive *vs*. active surveillance for safety) was retrieved.

For studies assessing efficacy, the main outcomes were the number of treated and infected participants (before and after treatment), CRs (the percentage of individuals who became helminth egg negative following treatment) and egg reduction rates (ERRs) (when available).

Number of AEs and specific reported symptoms (if detailed), type of AEs (*i*.*e*., symptom, observable or lab event) and whether AEs were associated with baseline parasite infection status were recorded for appraisal of safety data. If AE data were provided as number of participants with AEs and number of AEs, respectively, the earlier was preferred for data extraction.

### Statistical analysis

#### Analysis of efficacy aggregate data

For the meta-analysis, we used the open-source version of R (version 3.4.3.) using RStudio (version 1.0.143)[28]. Forest plots were performed using the forest command of R. Heterogeneity of datasets within the meta-analysis was quantified using *I*^*2*^ and random-effects models were performed to account for the heterogeneity between studies. The CRs and ERRs extracted from the selected publications were used to describe differences in efficacy performance. We used risk ratio’s (RRs) for the failure rate as an estimate of the true risk of being still infected after treatment in each treatment arm. A RR<1 indicates a lower risk of remaining infected when treated with the combination of ivermectin and albendazole compared to being treated with albendazole alone. A RR = 1 shows the risk of still being infected after treatment is the same in both groups. A RR>1 indicates that subjects in the albendazole alone group are at lower risk of still being infected.

#### Analysis of individual patient efficacy data

All individual patient data analyses were conducted using SAS system version 9.4 (SAS Institute, Cary, NC, United States of America). Individual patient’s egg counts at baseline and post-treatment were transformed in eggs per gram (EPG) of stool by multiplying the arithmetic mean (AM) number of eggs per Kato-Katz slide by 24. The AM EPG was calculated at baseline for each parasite species, study and treatment group within study.

Drug efficacy was expressed as ERR and CR. Individual ERR was calculated as the ratio of the difference between the pre- and post-treatment EPG to the pre-treatment EPG multiplied by 100 for each patient. Negative individual ERRs were classified as zero. The CRs and 95% binomial confidence intervals (CIs) were calculated as the percentage of STH infected individuals at baseline who turned negative at follow-up. The distribution of individual responses in egg excretion was expressed in centiles to quantitate the fraction of poor responders and plotted cumulatively. Both no change and an increase in EPG between pre- and post-treatment were considered as ERR = 0.

A linear model was used to evaluate the effects of treatments on the individual ERR for each STH (*A*. *lumbricoides*, *T*. *trichiura* and hookworm). The models accounted for clustering on study level and were adjusted for patient-level factors such as age, number of species observed in the stools and the log-transformed baseline EPG. Marginal means of ERR for each treatment were predicted from the model and compared pairwise with a Tukey adjustment for multiplicity. All tests were two-tailed; a p-value of 0.05 was deemed significant.

#### Analysis of aggregate safety data

Safety data from relevant publications were summarized using mainly descriptive statistics assessing frequencies of reported AEs and specific symptoms in co-treated participants, where applicable. The number of AEs per group and relative risk among studies comparing co-administered ivermectin and albendazole with single doses of albendazole or ivermectin alone were analysed applying a between-study meta-analysis as done for the efficacy data. Additionally, a sub-group meta-analysis was performed by further stratifying infection status of the respective study participants by parasitic disease (*i*.*e*. LF and/or onchocerciasis *vs*. STH). Studies not explicitly stating quantitative data on AEs and symptom reporting among co-treated individuals but providing valuable information on safety parameters were retained for qualitative appraisal.

## Results

### Literature search on efficacy data and study characteristics

**Fig 1:**
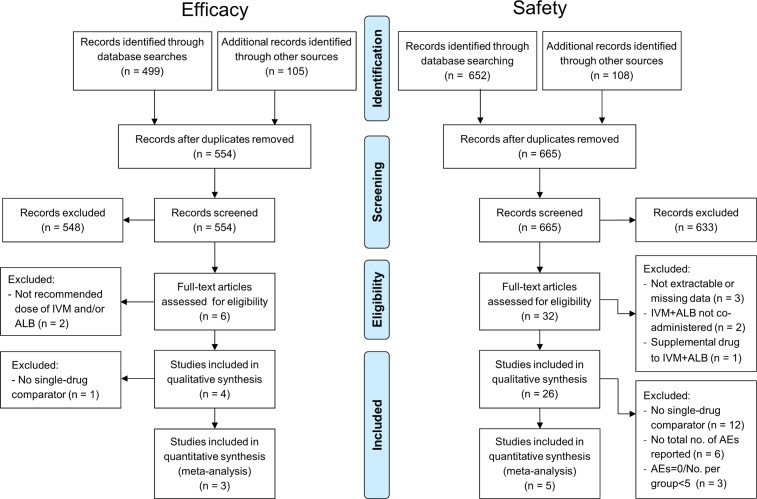
PRISMA flowchart showing the selection process of the efficacy and safety studies.

**Fig 2:**
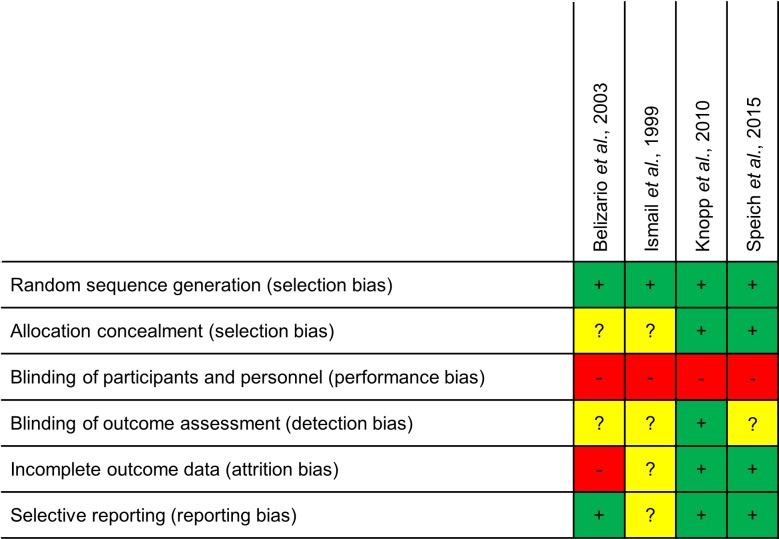
Quality assessment of included efficacy randomized controlled trials using the Cochrane criteria [26] for judging risk of bias. Note: + = low risk, - = high risk, ? = unclear.

Among the six potentially relevant studies identified, two [29, 30] were excluded because they did not use the recommended doses of ivermectin and/or albendazole (albendazole: 400 mg, ivermectin: 200 μg/kg) (Fig 1, Table A in S2 Text). As a result, we selected a total of four studies, of which one compared the co-administration of ivermectin-albendazole to albendazole and ivermectin alone [18], two compared the co-administration to albendazole alone [19, 20] and one compared ivermectin-albendazole to other therapies which were not considered in this review (Fig 1) [24]. The features and methodological quality of the selected studies are summarized in Fig 2. The more recent trials [20, 24] reported more methodological details on the study design and measures for mitigation of potential bias and thus reached higher quality levels than the older ones [18, 19].

Table 1 provides a brief overview on the four selected RCTs and their characteristics. Note that not all studies evaluated the efficacy of the drugs against all STHs: Ismail *et al*. [19] only assessed the efficacy against *T*. *trichiura* and Belizario *et al*. [18] did not evaluate efficacy against hookworms. The treatment outcome in all four selected studies was assessed between 7 and 39 days post-treatment (2/4 studies at +/- 21 days). This assessment was done by examining one or two stool samples. Two studies collected one sample, one of these studies does not report on how many slides were performed from the stool sample [19] and the other performed duplicate Kato-Katz thick smears [18]; the other two studies collected two samples and performed duplicate Kato-Katz thick smears on each one [20, 24].

**Table 1:** Treatments administered by each study and respective study characteristics for included efficacy studies.

Reference no.	Study	IVM + ALB	ALB alone (or with placebo)	IVM alone (or with placebo)	Country	Studied parasites	Follow-up period	Age group	No. stool samples
[18]	Belizario *et al*., 2003	X	X	X	Philippines	*T*. *trichiura* *A*. *lumbricoides*	7–14 days	6–12	1 at baseline 1 at follow-up
[19]	Ismail *et al*., 1999	X	X		Sri Lanka	*T*. *trichiura*	3 weeks	4–14	1 at baseline 1 at follow-up
[20]	Knopp *et al*., 2010	X	X		Tanzania	*T*. *trichiura* *A*. *lumbricoides* Hookworm	22–39 days	6–20	2 at baseline 2 at follow-up
[24]	Speich *et al*., 2015*	X			Tanzania	*T*. *trichiura* *A*. *lumbricoides* Hookworm	18–23 days	6–14	2 at baseline 2 at follow-up

Note: IVM + ALB = co-administration of ivermectin-albendazole, ALB = albendazole, IVM = ivermectin.

*Speich *et al*. 2015 [24] compared ivermectin-albendazole to albendazole-mebendazole, albendazole-oxantel pamoate and mebendazole alone.

Table 2 summarizes the efficacy outcomes of each single and the combined drug regimen investigated against the different STH species in all four studies. Outcome measures were CRs and ERRs–calculated using geometric means in all four studies. The first three studies in Table 2 show an improvement of the CR against *T*. *trichiura* when using the combination of ivermectin-albendazole *vs*. ivermectin or albendazole alone.

**Table 2:** Efficacy measures (*i*.*e*. cure rates (CRs) and egg reduction rates (ERRs)) by treatment arm against each soil-transmitted helminth species.

Study	Efficacy parameter	*T*. *trichiura*				*A*. *lumbricoides*				Hookworm			
		N	IVM + ALB (95% CI)	ALB alone (95% CI)	IVM alone (95% CI)	N	IVM + ALB (95% CI)	ALB alone (95% CI)	IVM alone (95% CI)	N	IVM + ALB (95% CI)	ALB alone (95% CI)	IVM alone (95% CI)
Belizario *et al*., 2003	CR	452	65%	32%	35%	306	78%	70%	78%	-	-	-	-
	ERR		100%	97%	98%		100%	100%	100%				
Ismail *et al*., 1999	CR	108	81%	44%	-	-	-	-	-	-	-	-	-
	ERR		95%	70%									
Knopp *et al*., 2010	CR	303	38% (29.8–46.4)	10% (5.4–16.3)	-	34	93%	100%	-	81	67%	59%	-
	ERR		91% (87.2–94.0)	40% (21.5–55.7)			100%	100%			96%	94%	
Speich *et al*., 2015*	CR	110	28% (19.0–36.0)	-	-	50	98% (94.0–100)	-	-	43	50% (34.2–65.8)	-	-
	ERR		95% (91.7–96.3)				100% (99.9–100)				96% (90.8–98.3)		

Note: IVM + ALB = co-administration of ivermectin-albendazole, ALB = albendazole, IVM = ivermectin. Not all studies provided 95% confidence intervals (CIs).

*Speich *et al*. 2015 [24] compared ivermectin-albendazole to albendazole-mebendazole, albendazole-oxantel pamoate and mebendazole alone.

### Comparison of efficacy outcomes between single-drug and combined treatment for each STH species

#### Efficacy against *T*. *trichiura* infections

**Fig 3:**
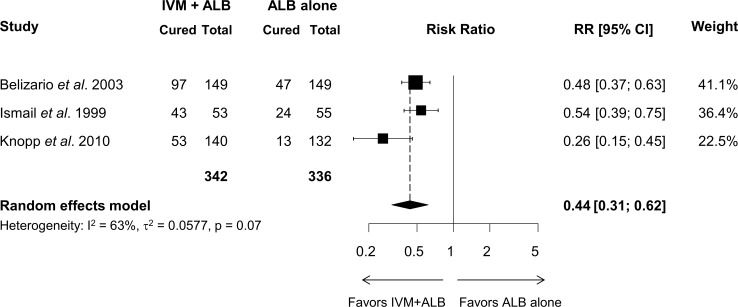
Forest plot displaying the results of a random-effects meta-analysis of aggregated data of the effect of the co-administration of ivermectin-albendazole on the number of patients infected with *T*. *trichiura* compared to albendazole alone.

The CRs and ERRs of the four studies evaluating the efficacy against *T*. *trichiura* are presented in Table 2. For the three studies comparing ivermectin-albendazole to albendazole alone (n = 342 and n = 336 patients, respectively), the aggregated-data meta-analysis is presented as a forest plot in Fig 3. The co-administration of ivermectin-albendazole was significantly more effective than albendazole alone at clearing *T*. *trichiura* infection, with a RR for still being infected post-treatment of 0.44 (95% CI = 0.31–0.62). While no bias indicators could be calculated since there were too few strata, all three studies favored the co-administration over single-agent albendazole. In the same three selected studies, patients treated with the co-administration had ERRs ranging from 91% to 100% for *T*. *trichiura* which is considerably higher than those for albendazole alone ranging from 40% to 97% (Table 2). Although they did not compare to either drug alone, Speich *et al*. (2015) [24] found a CR of 28% and an ERR of 95% using the co-administration of ivermectin and albendazole (Table 2). Belizario *et al*. [18] reported that the efficacy of the co-administration of ivermectin-albendazole was also higher than that of ivermectin alone (CR = 65% *vs*. CR = 35%, respectively) (Table 2).

**Fig 4:**
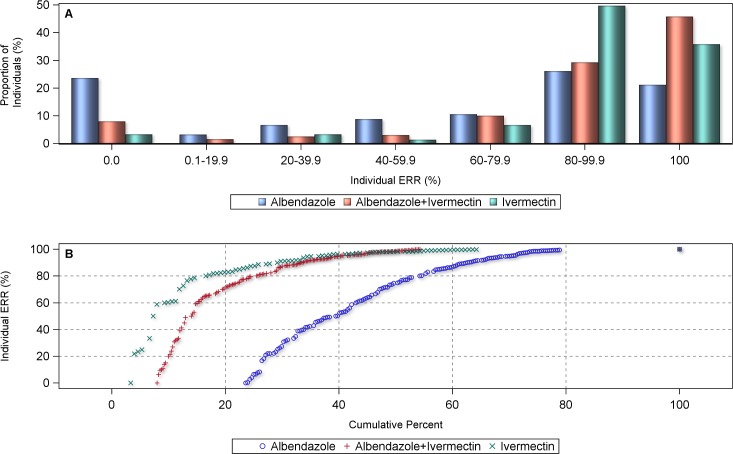
Proportional distribution (A) and cumulative centile curve (B) of individual egg reduction rates (ERRs) in *T*. *trichiura*-infected individuals (n = 845) by treatment arm.

The analysis of the individual patient data led to similar results, *i*.*e*. individual ERRs of 100% were significantly more often achieved with the co-administration of ivermectin and albendazole than with the administration of albendazole alone (mean individual ERR = 94.9%, 95% CI = 90.9–97.1% *vs*. mean individual ERR = 49.3%, 95% CI = 6.7–81.1%, respectively; p<0.05) (Fig 4). 78.9% of the *Trichuris*-infected individuals in the single albendazole arms from the pooled individual data sample revealed an ERR below 100%, compared to 54.2% of those in the co-administration as shown in the cumulative percentage curve. This would translate into a CR of 21.1% and 45.8%, respectively, for single and ivermectin-co-administered albendazole. The proportion of subjects who did not respond to treatment (ERR = 0) was 8% with the combination *vs*. 24% with albendazole alone. When the overall ERRs for each treatment arm were considered, both ivermectin alone (ERR = 72.1%) and ivermectin-albendazole co-administered (ERR = 84.5%), performed significantly better than albendazole alone (ERR = 59.1%) (p<0.001). The comparison of ivermectin alone and the combined treatment was significantly in favor of the latter (p<0.001). While ivermectin alone had slightly fewer ERRs of 0% than the ivermectin-albendazole combination treatment, the latter outperformed the single drug treatment with higher proportions of infection clearance (*i*.*e*. ERR = 100%).

#### Efficacy against *A*. *lumbricoides* infections

**Fig 5:**
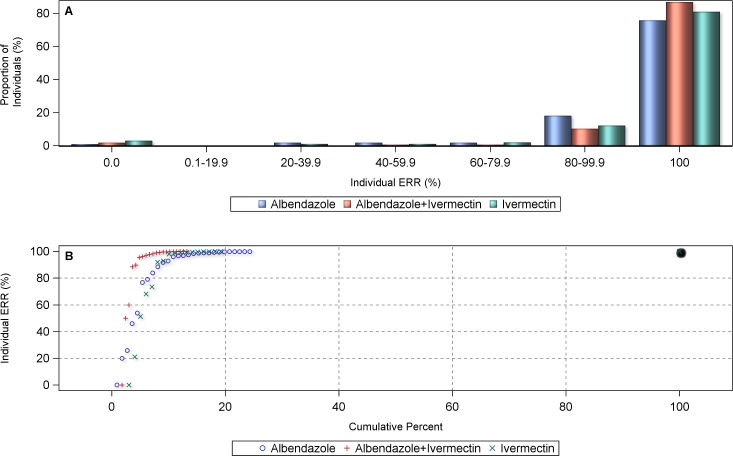
Proportional distribution (A) and cumulative centile curve (B) of individual egg reduction rates (ERRs) in *A*. *lumbricoides*-infected individuals (n = 385) by treatment arm.

As there were only two studies comparing the efficacy of the co-administration of ivermectin-albendazole *vs*. albendazole and ivermectin alone against *A*. *lumbricoides* infection, a meta-analysis could not be performed. Both studies showed comparable CRs among treatments and ERRs of 100% for both the single-agent and the combination arm. The individual patient data analysis also did not reveal any advantage of including ivermectin against *A*. *lumbricoides* (Fig 5) with summary ERR estimates of 98.4% (albendazole, 95% CI = 93.8–100.0%) *vs*. 98.6% (ivermectin-albendazole, 95% CI = 94.7–100.0%) (p = 0.993).

#### Efficacy against hookworm infections

**Fig 6:**
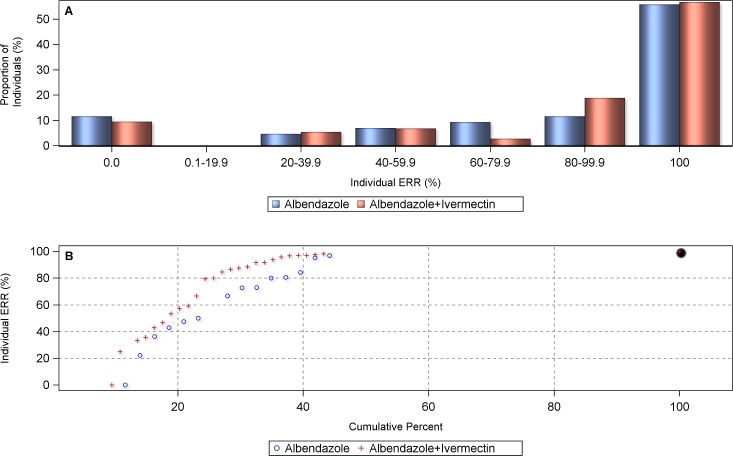
Proportional distribution (A) and cumulative centile curve (B) of individual egg reduction rates in hookworm-infected individuals (n = 117) by treatment arm.

A single study compared the efficacy of ivermectin-albendazole *vs*. albendazole alone against hookworm infections [20]. The co-administration produced an only marginally higher CR and a similar ERR than albendazole alone (Table 2) [20]. The individual patient data analysis, that also included the hookworm infections treated with the co-administration from the study by Speich *et al*. (2015) [24] did not reveal any significant difference in efficacy between the two treatments against hookworm with ERRs of 81.7% *vs*. 78.0%, respectively (p = 0.643) (Fig 6).

### Results from the literature search on safety data and study characteristics

A total of 665 records were identified using the described search strategy (see S1 Text). These articles were screened for safety data on the co-administration of albendazole and ivermectin. In total, 32 studies were retained and full-text articles checked on eligibility criteria as defined above (see Fig 1). Details on study characteristics and reasons for inclusion or exclusion are provided as a supplementary file (Table B in S2 Text). Six studies were excluded due to non-extractability of the data or missing relevant information (n = 3) [18, 31, 32], consecutive instead of co-administration of ivermectin and albendazole (n = 2) [16, 33] and one study additionally administered diethylcarbamazine (DEC) together with the ivermectin-albendazole combination (n = 1) [34]. A total of 26 studies were included and considered for quantitative or qualitative appraisal in this review. Among these, 24 provided quantitative information on AEs (Table 3) of which 22 detailed specific information on symptoms (Table 4). One review [35] served as a supplementary quantitative and qualitative information source to complement data from included original research articles. Another study provided description of AEs with regard to pregnancy outcome [36]. All studies with quantitative data that provided the actual number of AEs, the total number of treated individuals in the co-administered ivermectin-albendazole group and that had at least one single drug comparator (albendazole or ivermectin) group were selected for further analysis by means of meta-analysis (n = 5) (Table 3). Studies with zero AEs in all groups [37, 38] or less than five individuals per treatment arm [39] were not considered.

**Table 3:** Studies reporting quantitative data for adverse events (AEs) after ivermectin-albendazole co-administration (n = 24).

Reference no.	Study	Study type	Country	Disease (parasite)	IVM+ALB		IVM		ALB	
					No. treated	No. AEs	No. treated	No. AEs	No. treated	No. AEs
[43]	Addis *et al*., 1997	Trial (RCT)	Haiti	wb	44	NR	43	NR	27	NR
[44]	Amsden *et al*., 2007	Trial (open)	USA	healthy	18	1	-	-	-	-
[45]	Anto *et al*., 2011	Trial (matched)	Ghana	sh, sm, (wb, onc)	15552	130	-	-	-	-
[37]	Asio *et al*., 2009a	Trial (matched)	Uganda	mp	15	0	15	0	13	0
[38]	Asio *et al*., 2009b	Trial (RCT)	Uganda	mp	86	0	96	0	-	-
**[40]**	**Awadzi *et al*., 2003**^**$**^	**Trial (RCT)**	**Ghana**	**onc**	**14**	**14**	**14**	**11**	**14**	**13**
[46]	Coulibaly *et al*., 2015	prospective cross-sectional	Mali	wb	2135	13	-	-	-	-
[47]	Dembele *et al*., 2010^†^	Trial (RCT)	Mali	wb, mp	42	9	-	-	-	-
**[41]**	**Dunyo *et al*., 2000**	**Trial (RCT)**	**Ghana**	**wb**	**332**	**47**	**295**	**36**	**336**	**31**
[48]	Hodges *et al*., 2010	MDA post-treatment reporting	Sierra Leone	wb	1104407	146	-	-	-	-
[49]	Ismail *et al*., 1998	Trial (blinded)	Sri Lanka	wb	13	NR	-	-	12	NR
[50]	Ismail *et al*., 2001^#^	Trial (blinded)	Sri Lanka	wb	31	NR	-	-	-	-
[51]	Keiser *et al*., 2003	Trial (blinded)	Mali	wb, mp	40	11	-	-	-	-
**[20]**	Knopp *et al*., 2010*	**Trial (RCT)**	**Tanzania (Unguja island)**	**tri**	**144**	**64**	**-**	**-**	**136**	**60**
**[42]**	**Makunde *et al*., 2003**	**Trial (RCT)**	**Tanzania (mainland)**	**wb, onc**	**20**	**11**	**-**	**-**	**13**	**5**
[52]	Na-Bangchang *et al*., 2006	Trial (open)	Thailand	healthy	23	0	-	-	-	-
**[30]**	**Ndyomugyenyi *et al*., 2008**	**Trial (RCT)**	**Uganda**	**sth**	**199**	**8**	**198**	**24**	**194**	**16**
[39]	Shenoy *et al*., 1999	Trial (open)	India	bm	16	12	-	-	3	2
[53]	Shenoy *et al*., 2000	Trial (open)	India	bm	12	6	-	-	-	-
[54]	Simonsen *et al*., 2004	Trial (RCT)	Tanzania (mainland)	wb	586	NR	-	-	635	NR
[24]	Speich *et al*., 2015	Trial (RCT)	Tanzania (Pemba island)	tri	108	22	-	-	-	-
[55]	Tafatatha *et al*., 2015	Trial (RCT)	Malawi	wb	70	22	-	-	-	-
[56]	Turner *et al*., 2006	Trial (RCT)	Ghana	wb, wbb	28	20	-	-	-	-
[57]	WHO, 2003	MDA post-treatment reporting	Burkina Faso, Nigeria, Tanzania (Mafia & Zanzibar islands)	lf	9831	2358	-	-	-	-

^$^No. of AEs based on positive vs. zero Mazzotti reaction scores.

^†^Two different schemes of IVM/ALB combination: 150 μg/kg+400 mg (n = 22) and 400 μg/kg+800 mg (n = 20)

^#^Two different schemes of IVM/ALB combination: 200 μg/kg+400 mg (n = 16) and 400 μg/kg+600 mg (n = 15).

*No. of AEs instead of no. of participants with AEs.

Disease (parasite) abbreviations: bm = *Brugia malayi*, lf = lymphatic filariasis (species not specified), mp = *Mansonella perstans*, onc = *Onchocerca volvulus*, sh = *Schistosoma haematobium*, sm = *Schistosoma mansoni*, sth = soil-transmitted helminths, tri = *Trichuris*, wb = *Wuchereria bancrofti*, wbb = *Wolbachia* bacteria

RCT = randomized controlled trial, NR = not reported: no overall number of patients with AEs or AEs itself provided, AE frequencies only given for specific symptoms.

Studies in bold were subjected to meta-analysis.

**Table 4:** Frequencies of AEs and symptoms assessed after co-administration of ivermectin and albendazole and type of AE data provided.

Study (1st authμor + year)			Addiss 1997 [43]	Amsden 2007 [44]	Anto 2011 [45]	Asio 2009a^α^ [37]	Awadzi 2003 [40]	Dembele 2010^β^ [47]	Dunyo 2000 [41]	Hodges 2010 [48]	Ismail 1998 [49]	Ismail 2001^γ^ [58]	Keiser 2003 [51]	Knopp 2010 [20]	Makunde 2003 [42]	Na-Bangchang 2006 [52]	Ndyomugyenyi 2008^δ^ [30]	Shenoy 1999 [39]	Shenoy 2000 [53]	Simonsen 2004 [54]	Speich 2015^ε^ [24]	Tafatatha 2015 [55]	Turner 2006 [56]	WHO 2003^ζ^ [57]
**Dosage**																								
Ivermectin (in μg/kg)			200–400	200–400	height	150–200	2x6mg tablets	150 400	150–200	MDA dose	400	200 400	200	200	150	200	height	200	200	150–200	200	200–400	150	150–250
Albendazole (in mg)			400	400	MDA dose	400	400	400 800	400	MDA dose	600	400 600	400	400	400	400	400	400	400	400	400	400–800	400	400
**Time span of follow-up** (if active surveillance)			3–5 days	7 days	passive	7 days	30 days	7 days	5 days	5 days	4x/day for 48h	5 days	5 days	48h	4x/day for 48h	8 days	passive	7 days	5 days	5 days	3h and 24h	7 days	48h	5–7 days
**N (treated with IVM-ALB)**			44	18	15552	15	14	a) 22 b) 20	332	1104407	13	a) 16 b) 15	40	144	27	23	199	16	12	586	108	70	28	9831
**AEs by grade**																								
	Mild		OBS	1 (5.6%)	130 (0.84%)	0 (0.0%)	-	a) 1 (4.5%) b) 3 (15.0%)	45 (13.6%)	146 (0.01%)	-	-	11 (27.5%)	32 (22.2%)	9 (33.3%)	0 (0.0%)	8 (4.0%)	12 (75%)	6 (50.0%)	OBS	22 (20.4%)	OBS	17 (63%)	1289 (13.1%)
	Moderate		OBS	0 (0.0%)	0 (0.0%)	0 (0.0%)	-	a) 2 (9.1%) b) 1 (5.0%)	2 (0.6%)	0 (0.0%)	1 (7.7%)	-	0 (0.0%)	32 (22.2%)	2 (7.4%)	0 (0.0%)	0 (0.0%)	0 (0.0%)	0 (0.0%)	0 (0.0%)	0 (0.0%)	OBS	3 (11.1%)	918 (9.3%)
	Severe		OBS	0 (0.0%)	0 (0.0%)	0 (0.0%)	-	0 (0.0%)	0 (0.0%)	1 (0.0%)	0 (0.0%)	0 (0.0%)	0 (0.0%)	0 (0.0%)	0 (0.0%)	0 (0.0%)	0 (0.0%)	0 (0.0%)	0 (0.0%)	0 (0.0%)	0 (0.0%)	0 (0.0%)	0 (0.0%)	151 (1.5%)
**Gastrointestinal symptoms**																								
	Epigastric/abdominal pain or discomfort		OBS	1 (5.6%)	11 (0.07%)	pre-treat	-	a) 0 (0.0%) b) 2 (10.0%)	4 (1.1%)	10 (0.0%)	-	-	-	21 (14.6%)	-	0 (0.0%)	4 (2%)	-	-	-	13 (12.0%)	OBS	-	4.4%
	Diarrhoea		OBS	-	4 (0.03%)	0 (0.0%)	-	a) 0 (0.0%) b) 1 (5.0%)	4 (1.1%)	-	-	-	1 (2.5%)	4 (2.8%)	-	0 (0.0%)	-	-	-	OBS	3 (2.8%)	-	-	3.5%
	Vomiting		OBS	-	4 (0.03%)	0 (0.0%)	-	-	-	9 (0.0%)	-	-	3 (7.5%)	3 (2.1%)	-	0 (0.0%)	0 (0.0%)	-	-	OBS	1 (0.9%)	-	-	-
	Nausea		-	-	8 (0.05%)	0 (0.0%)	-	-	-	-	-	-	-	11 (7.6%)	-	0 (0.0%)	-	-	-	-	2 (1.9%)	-	-	4.4%
	Anorexia/loss of appetite		-	-	2 (0.01%)	-	-	-	-	-	OBS^η^	-	1 (2.5%)	-	-	-	0 (0.0%)	-	-	-	-	-	-	-
	Blood in stool		-	-	5 (0.03%)	-	-	-	-	-	-	-	-	-	-	-	-	-	-	-	-	-	-	-
	Bitter taste		-	-	-	-	-	-	-	-	-	-	-	-	-	0 (0.0%)	-	-	-	-	-	-	-	-
**CNS and musculoskeletal symptoms**																								
	Headache		28 (63.6%)	-	13 (0.08%)	pre-treat	6 (42.9%)	-	17 (4.6%)	16 (0.0%)	OBS	OBS	11 (27.5%)	5 (3.5%)	0 (0.0%)	0 (0.0%)	0 (0.0%)	OBS	OBS	14 (2.4%)^κ^	5 (4.6%)	OBS	OBS	7.7%
	Fever		28 (63.6%)^θ^	-	7 (0.05%)	pre-treat		-	20 (5.4%)	-	OBS	OBS	5 (12.5%)	6 (4.2%)	5 (18.5%)	0 (0.0%)	4 (2%)	OBS	OBS	9 (1.5%)^κ^	3 (2.8%)	OBS	OBS	3.5%
	Fatigue/tiredness/lethargy		-	-	9 (0.06%)	pre-treat	-	-	-	-	OBS^η^	-	3 (7.5%)	4 (2.8%)	0 (0.0%)	0 (0.0%)	-	OBS	-	-	3 (2.8%)	-	-	3.5%
	Dizziness		-	-	10 (0.06%)	pre-treat	-	-	-	13 (0.0%)	-	-	-	-	0 (0.0%)	0 (0.0%)	-	-	-	OBS	-	-	-	3.4%
	Shivering/chills		-	-	-	-	-	-	-	-	OBS^η^	-	-	3 (2.1%)	0 (0.0%)	-	-	OBS	OBS	-	-	-	OBS	-
	Body weakness		-	-	6 (0.04%)	-	-	-	9 (2.4%)	-	OBS	OBS	-	-	-	-	-	-	-	-	-	-	-	-
	Vertigo		-	-	-	-	-	-	-	-	-	-	-	2 (1.7%)	-	-	-	-	-	-	2 (1.9%)	-	-	-
	Diaphoresis/excessive sweating			-	-	-	-	-	-	-	0 (0.0%)^η^	-	-	-	-	-	-	-	-	-	-	-	-	-
	Myalgia/muscle pain		15 (34.1%)	-	11 (0.07%)	pre-treat	-	-	16 (4.3%)	-	OBS	OBS	2 (5%)	-	0 (0.0%)	-	-	OBS	OBS	-	-	-	-	4.6%
	Arthralgia/joint pain		-	-	8 (0.05%)		-	-		-	OBS^η^	-	-	-	-	-	-	-	-	OBS	-	OBS	-	
	Lumbar/lower back pain		-	-	-	-	-	-	-	-	-	-	-	-	-	0 (0.0%)	-	-	-	-	-	-	-	-
	Numbness of limbs		-	-	-	-	-	-	-	1 (0.0%)	-	-	-	-	-	-	-	-	-	-	-	-	-	-
**Allergic-type/immune response-related symptoms**																								
	Itching/pruritis		-	-	10 (0.06%)	-	-	-	3 (0.8%)	79 (0.01%)	-	-	-	2 (1.7%)	4 (14.8%)	-	2 (1%)	-	-	OBS	-	-	OBS	-
	Rashes		-	-	4 (0.03%)	0 (0.0%)	-	-	2 (0.5%)	25 (0.0%)	-	-	1 (2.5%)	-	-	-		-	-	-	-	-	OBS	-
	Urticaria		-	-	-	-	-	a) 1 (4.5%) b) 0 (0.0%)	-	-	-	-	-	3 (2.1%)	-	-	-	-	-	-	-	-	-	-
	Oedema/swelling		-	-	-	-	-	a) 2 (9.1%) b) 1 (5.0%)	-	59 (0.01%)	-	-	-	-	1 (3.7%)	-	-	-	-	-	-	-	-	-
	Swelling of the limbs		-	-	4 (0.03%)	0 (0.0%)	-	-	-	-	-	-	-	-	-	-	-	-	-	-	-	-	-	0.5%
	Swelling of the face		-	-	3 (0.02%)	0 (0.0%)	-	-	-	-	-	-	-	-	-	-	-	-	-	-	-	-	-	0.1%
	Tender or swollen lymph nodes/adenitis		-	-	2 (0.01%)	-	OBS	-	-	-	-	-	-	-	1 (3.7%)	-	-	0 (0.0%)	0 (0.0%)	-	-	-	-	-
	„String sign”(dilated painful/inflamed lymphatic channels)		-	-	-	-	-	-	-	-	-	-	-	-	-	-	-	0 (0.0%)	0 (0.0%)	-	-	-	-	-
	Nodules (scrotal)		0 (0.0%)	-	-	0 (0.0%)	-	-	-	-	0 (0.0%)	0 (0.0%)	-	-	-	-	-	0 (0.0%)	0 (0.0%)	-	-	-	-	-
	Mazzotti-type toxicity (combination of AEs)^λ^		-	-	-	0 (0.0%)	2 (13.3%)	-	-	-	-	-	-	-	-	-	-	-	-	-	-	-	-	-
	Allergic reaction		-	-	-	-	-	-	-	-	-	-	-	-	-	-	-	-	-	-	2 (1.9%)	-	-	-
	Axillary abscess		-	-	-	-	-	-	-	-	-	-	-	-	-	0 (0.0%)	-	-	-	-	-	-	-	-
**Long term side effects (including hematologic, metabolic, endocrine and cardiovascular indicators)**																								
	Liver function abnormalities	ALT	-	0 (0.0%)	-	-	0 (0.0%)^μ^	-	-	-	5 (38.5%)	a) 2 (12.5%) b) 1 (6.7%)	-	-	-	OBS	-	-	-	-	-	-	-	-
		AST	-	0 (0.0%)	-	-	0 (0.0%)^μ^	-	-	-			-	-	0 (0.0%)	OBS	-	-	-	-	-	-	-	-
		tot/dir bilirubin	-	0 (0.0%)	-	-	0 (0.0%)^μ^	-	-	-			-	-	0 (0.0%)	OBS	-	-	-	-	-	-	-	-
	Kidney function abnormalities/creatinine		-	0 (0.0%)	-	-	0 (0.0%)^μ^	-	-	-	0 (0.0%)	a) 2 (12.5%) b) 1 (6.7%)	-	-	0 (0.0%)	-	-	-	-	-	-	-	-	-
	Leucopenia/reduced white blood cell count		-	0 (0.0%)	-	-	0 (0.0%)^μ^	-	-	-	0 (0.0%)	-	-	-	0 (0.0%)	-	-	-	-	-	-	-	-	-
	Proteinuria		-	0 (0.0%)	-	-	0 (0.0%)^μ^	-	-	-	0 (0.0%)	-		-	-	OBS	-	-	-	-	-	-	-	-
	Hematuria		-	0 (0.0%)	-	-	0 (0.0%)^μ^	-	-	-	1 (7.7%)	-	-	-	-	-	-	-	-	-	-	-	-	-
	Polyuria		-	-	-	-	-	-	-	6 (0.0%)	-	ππ-	-	-	-	-	-	-	-	-	-	-	-	-
	Abnormal ECG/heart rate		-	0 (0.0%)	-	-	0 (0.0%)^μ^	-	-	-	0 (0.0%)	-		-	0 (0.0%)	0 (0.0%)	-	-	-	-	-	-	-	-
	Low blood pressure (syncope or hypotension)		-	-	-	-	-	-	-	-	-	-	0 (0.0%)	-	0 (0.0%)	0 (0.0%)	-	0 (0.0%)	0 (0.0%)	-	-	-	-	-
	Tachycardia/palpitations		-	-	-	-	-	-	-	-	-	-	-	-	4 (14.8%)	0 (0.0%)	-	-	-	-	-	-	-	-
	Erectile dysfunction		-	-	-	-	-	-	-	3 (0.0%)	-	-	-	-	-	-	-	-	-	-	-	-	-	-
**Ocular symptoms**																								
	Reddening of the eyes/conjunctivitis		-	-	8 (0.05%)	-	1 (7.1%)	-	-	-	-	-	-	-	-	-	-	-	-	-	-	-	-	-
	Eye pain and lacrimation		-	-	-	-	2 (13.3%)	-	-	-	-	-	-	-	-	-	-	-	-	-	-	-	-	-
**Respiratory symptoms**																								
	Cough, not with cold		19 (43.2%)	-	-	-	-	-	-	-	0 (0.0%)^ι^	-	0 (0.0%)	-	-	-	-	-	-	-	-	-	-	-
	Dyspnea/wheezing		OBS	-	-	-	-	-	-	-	1 (7.7%)	-	2 (5%)	-	-	-	-	-	-	-	-	-	-	-
	Sore throat		-	-	1 (0.006%)	-	-	-	-	-	-	-	-	-	-	-	-	-	OBS	-	-	-	-	-
	Running nose		-	-	-	-	-	-	-	-	-	-	-	-	-	0 (0.0%)	-	-	-	-	-	-	-	-
	Nasal congestion		-	-	-	-	-	-	-	-	-	-	-	-	-	0 (0.0%)	-	-	-	-	-	-	-	-
**Type of assessed AE data**																								
	Symptom events		x	x	x	x	x	x	x	x	x	x	x	x	x	x	x	x	x	x	x	x	x	x
	Measurable/Observable (clinical examination)		x	x	x	x	x	x	x		x	x	x	x	x	x	x	x	x	x	x		x	
	Lab events			x			x				x	x			x	x		x						
	Day 0 data^ν^			x (=)		x (=)	x (↑)				x (↑)		x^ξ^		x (=)	x (=)					x (↓)		x (↑)	
	AE reporting related with infection status or intensity (parasitic disease)		x (lf)				x (onc)		x (lf)		x (lf)	x (lf)	x (lf)							x (lf)		x (lf)	x (lf)	

Abbreviations: AE = adverse event, ALT = alanine aminotransferase, AST = aspartate aminotransferase, CNS = central nervous system, ECG = electrocardiogram, lf = lymphatic filariasis, MDA = mass drug administration, - = not reported/detailed, OBS = observed but no quantitative data per treatment arm provided, onc = onchocerciasis

α No AEs found, no increase in infection-related symptoms as assessed pre-treatment (pre-treat)

β Study including two different IVM-ALB co-administered treatment arms: a) 150 μg/kg ivermectin + 400 mg albendazole, b) 400 μg/kg ivermectin + 800 mg albendazole

γ Study including two different IVM-ALB co-administered treatment arms: a) 200 μg/kg ivermectin + 400 mg albendazole, b) 400 μg/kg ivermectin + 600 mg albendazole

δ Study with pregnant women

ε Missing information completed from data provided from personal communication

ζ Data completed with information provided in WHO 2005 [59]

η Data completed with information provided in Horton *et al*. 2000 [35]

θ Self-reported

κ Reconstructed from chi-square test results provided for comparison between treatment groups

λ The Mazzotti reaction includes: dermal (e.g., pruritis, lesions, oedema), ocular (e.g. conjunctivis, uveitis), lymphatic (adenitis, lymphoedema), cardiovascular (e.g., hypotension, tachycardia), respiratory, musculosceletal (e.g., myalgia, arthralgia) and other systemic (e.g., fever) manifestations [60]

μ Details on type of laboratory assessments provided in Awadzi et al. 1995 [61]

ν Interpretation of day 0 data: no significant difference in pre- and post-treatment frequency of AEs (symptoms or clinical indicators) (=), increased frequency of AEs after treatment (↑), and decreased frequency of AEs after treatment (↓)

ξ Pre-treatment questionnaire data used to define exclusion criteria (subjects with known lf post-treatment symptoms were excluded)

**Fig 7:**
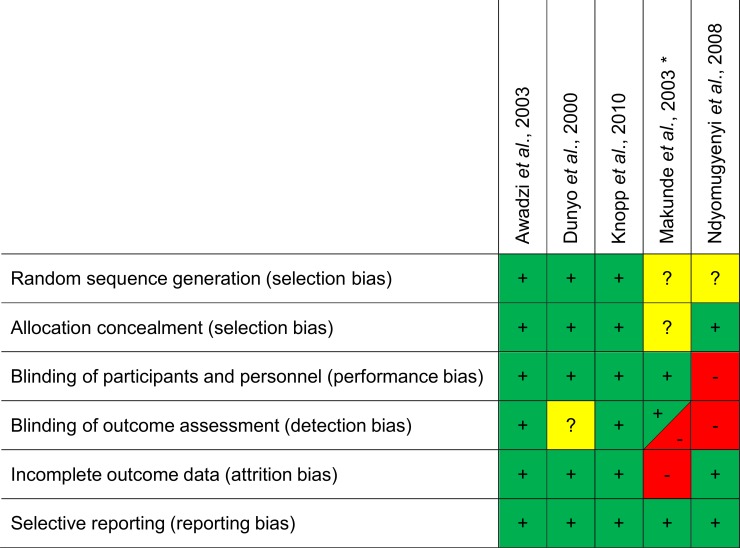
Quality assessment of included safety studies using the Cochrane criteria for judging risk of bias. Note: + = low risk,— = high risk, ? = unclear.* Study including two groups with different designs: *W*. *bancrofti*-single-infected group = open design, *W*. *bancrofti*/*O*. *volvulus*-co-infected group = double blind design.

The quality and types of potential biases of these five studies is summarized in Fig 7. Two studies reached the highest quality grading [20, 40], while one did not clearly state about blinding of outcome assessors [41] and two studies followed an open-label or only partly blinded study design [30, 42], provided incomplete information on random allocation measures and thus reached lower grading.

Among the included safety studies providing original quantitative information on treated subjects (n = 24), the number of monitored individuals after treatment administration varied considerably and mainly depended on study type and design (Table 3, Table B in S2 Text). Twenty one studies were clinical trials of which the majority (n = 16) applied an active surveillance approach and more than half (n = 12) were RCTs. Four studies reported safety parameters assessed either using an observational [46, 48, 57] or a trial design (including comparison between matched groups) [45] embedded in regional or national control programs applying mass drug administration (MDA) against LF. Thus, these four studies had much larger sample sizes.

Within the trials, most studies involved participants with filariasis such as LF due to *Wuchereria bancrofti* (n = 7) [41, 43, 49, 50, 54–56] or *Brugia malayi* (n = 2) [39, 53], onchocerciasis due to *Onchocerca volvulus* (n = 1) [40], mansonellosis due to *Mansonella perstans* (n = 2) [37, 38] or co-infections of the above (n = 3) [42, 47, 51]. Three studies assessed the safety of ivermectin-albendazole co-administration in patients infected with STHs [20, 24, 30] and one study in patients with schistosomiasis caused by *Schistosoma haematobium* and/or *S*. *mansoni* [45]. Two studies assessed the safety of co-administered ivermectin and albendazole in healthy subjects [44, 52]. Trials were conducted in ten different countries whereas observational data after MDA campaigns was available for 5 countries. Of the 21 trials, eight were from East Africa (Malawi, Tanzania and Uganda), seven from West Africa (Ghana and Mali), five from South-East Asia (India, Sri Lanka and Thailand), one from Latin America and the Caribbean (Haiti) and one from North America (USA). Post-MDA-treatment safety reporting was available for West Africa (Burkina Faso, Mali, Nigeria and Sierra Leone) and East Africa (Tanzania).

#### Most common adverse events

Table 4 provides a detailed overview on all different types of AEs reported after co-administration of ivermectin and albendazole. There is a marked imbalance in terms of population size and reporting methodology between the two observational studies (which followed up 1,104,407 and 9831 patients treated with the combination therapy respectively) [48, 57] and the other studies (which collectively enrolled 17,314 patients). The latter only reported AEs for the co-administration of ivermectin-albendazole, which were mild or moderate in severity. The observational studies on the other hand reported 1 and 151 severe AEs, respectively, but none was considered as a serious adverse event (SAE). Headache, fever, abdominal pain, muscle/joint pain and allergic reactions like pruritus and rashes ranked among the top five symptoms assessed by these studies. Fever and headache were the most frequent AEs. Abdominal pain was prominently reported in trials of intestinal helminthiases [20, 24, 30, 45] and muscle/joint pain together with skin reactions (e.g. pruritus and rashes) were more often observed in studies of filariasis [41–43, 48]. Laboratory events were rarely reported; there were altogether 8 cases of increased levels of liver enzymes [49, 52, 58] that usually returned to normal levels within few weeks.

#### Safety of ivermectin-albendazole compared to albendazole alone

**Fig 8:**
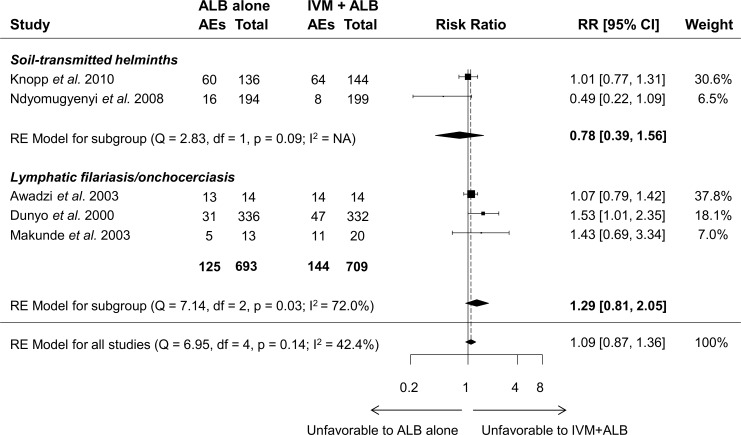
Forest plots showing random-effects meta-analysis of the number of patients with adverse events (AEs) after co-administration of ivermectin-albendazole compared to albendazole alone stratified by helminthic disease. RE = random effects. NA = not applicable, RE = random effects.

Five RCTs compared the safety of co-administered ivermectin and albendazole to albendazole alone. Fig 8 shows the results from the random-effects meta-analysis from all studies pooled as well as stratified by parasitic infection type (filariasis *vs.* soil-transmitted helminthiasis). The overall estimate shows an RR of 1.09 (95% CI = 0.87–1.36) for AEs in the co-administration group *vs*. albendazole alone. When stratified by helminthic disease, the RR for patients with filariasis (*i*.*e*., LF and onchocerciasis) was 1.29 (95% CI = 0.81–2.05), and 0.78 (95% CI = 0.39–1.56) for STH in co-treated compared to albendazole-alone treated patients. None of these comparisons was statistically significantly different.

#### Safety of ivermectin-albendazole compared to ivermectin alone

**Fig 9:**
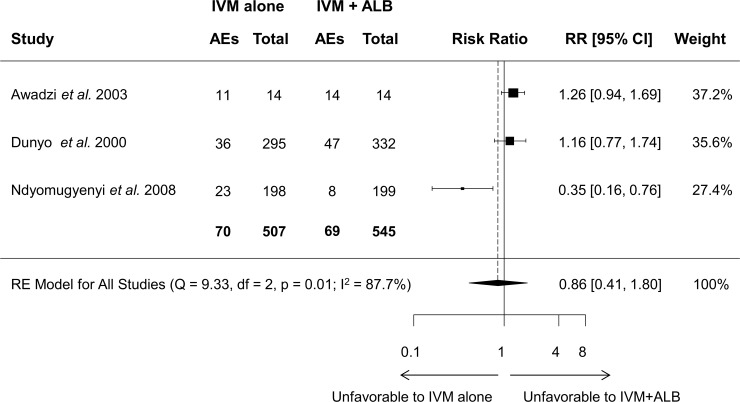
Forest plot showing random-effects meta-analysis of the number of patients with adverse events after co-administration of ivermectin-albendazole compared to ivermectin alone.

Three of the five studies included in the meta-analysis also compared the co-administration to single ivermectin administration, with inconsistent results: Awadzi *et al*. [40] and Dunyo *et al*. [41] reported slightly more AEs in the combination arm, while the opposite result was demonstrated by Ndyomugyenyi *et al*. [30]. Consequently, no statistical difference in the number of AEs was found in the meta-analysis model (RR = 0.86, 95% CI = 0.41–1.80) as shown in Fig 9.

#### Additional outcomes and factors associated with safety

Two studies explored the relationship between co-administration of ivermectin and albendazole and pregnancy outcome. There was no evidence of a higher risk of congenital malformation or abortions (spontaneous or miscarriages) due to treatment. Moreover, there was no significant association between treatment and the proportion of newborns with low birth weight or congenital abnormalities and the number of stillbirths [30, 36].

No difference in safety was observed between low- and high dose ivermectin-albendazole formulations within the same trials [47, 55, 58].

As highlighted in Table 4, nine studies observed direct relationships between the baseline microfilariae levels and the number and intensity of AEs. Of these, eight were related to *W*. *bancrofti* infections and one to *O*. *volvulus*. This latter study used the Mazzotti score to distinguish between the reaction to the killing of microfilariae and direct drug-related AEs [40].The relationship between parasite death and number of AEs is further supported by the two studies assessing the efficacy and safety in *M*. *perstans* infection. The combination therapy showed no effect on *Mansonella* microfilariae levels and not a single AE was reported [37, 38].

Finally, study design and the method of assessing safety (e.g. active *vs*. passive reporting) may also influence the extent of reported, observed or measured events. Passively assessed target populations revealed lower numbers of AEs compared to intensively monitored participants within RCTs or large-scale active surveillance reports (Table 4). Furthermore, it is important to take into account the baseline symptomatology or clinical indicators such as hematological or metabolic parameters when symptom reports or laboratory events are considered for AE assessment (treatment-emergent adverse events, TEAE). Nine studies considered any kind of pre-treatment parameters (e.g. self-reported symptoms or clinical indicators). Of these, three showed an increase from baseline values [40, 49, 56], four reported no difference [37, 42, 44, 52], one only used the pre-treatment parameters as exclusion criteria [51] and one study observed reduced symptom reporting compared to baseline in the ivermectin-albendazole co-administration arm [24].

## Discussion

Infections with STH continue to be amongst the most common infections worldwide and PC is the main strategy applied for morbidity control [4]. PC relies mainly on two benzimidazole drugs (albendazole and mebendazole) but they are not equally efficacious against all three helminths; *T*. *trichiura* remains the main challenge [8, 62].

To our knowledge, this is the first systematic review and meta-analysis exploring the efficacy and safety of the co-administration of ivermectin and albendazole against STH infections. Both our aggregate and individual patient data analysis indicate that the co-administration of ivermectin and albendazole is more effective against *T*. *trichiura* than either of the drugs alone. The co-administration does not seem to offer advantages over albendazole alone on either hookworm or *A*. *lumbricoides*.

The inclusion of ivermectin-albendazole co-treatment in the WHO Model List of Essential Medicines to treat STH is likely to promote its roll-out in future MDA campaigns [21]. From our findings one can, therefore, anticipate that this will provide enhanced efficacy in *T*. *trichiura-*infected individuals (the most difficult-to-treat STH infection [8, 62]). Although not evaluated in the present review, the albendazole-ivermectin co-administration would also benefit *Strongyloides stercoralis*-infected individuals [63, 64]. On the other hand, the decrease of PC for LF represents a risk of losing ancillary benefit for soil-transmitted helminthiasis control in the framework of the Global Programme to Eliminate Lymphatic Filariasis (GPELF). In more detail, in 2015, 52 million school-aged children received albendazole-ivermectin in this program in STH co-endemic areas [10]. With decreasing PC for LF, effective transitions are required to cover the lost contribution of the GPEFL program for STH control.

It is worth highlighting that coverage and compliance of target populations are essential for the successful future roll-out of integrated treatment of ivermectin and albendazole against STH infections. Knowledge about MDA programs and its efficacy against the target disease together with appropriate handling of potential safety issues by MDA implementation staff has been shown to positively influence the target populations’ acceptance of ivermectin-albendazole treatment campaigns against LF [65]. However, LF programs only target children aged five years or above meaning all preschool-aged children have not been receiving ivermectin-albendazole. As the official target age group of STH control includes preschool-aged children, it would be of great value to study both efficacy and safety of this combination in this age group. Of note, a recent study evaluated for the first time the efficacy and safety of ivermectin in preschoolers infected with *T*. *trichiura* and demonstrated that the drug can be safely used in young children [17].

With more than 20 studies reporting safety-relevant information, this systematic review helps to better define the tolerability profile of the co-administration of ivermectin and albendazole covering not only soil-transmitted helminthiasis but also filariasis. Overall, the co-administration was well-tolerated and caused only mild and transient AEs. While study design, size and methods for assessing safety varied across these studies, collectively they covered a range of patient populations (including adult men, pregnant women and schoolchildren) and diseases (from healthy individuals to those with soil-transmitted helminthiasis or filariasis).

When considering the five RCTs comparing single- and co-treated patients, we found no difference in the incidence of AEs between ivermectin plus albendazole *vs*. albendazole or ivermectin alone. Of note, among the five studies included in our review, two were on STH treatment [20, 30] and three on another indication [41–43]; when stratifying by type of infection, we found no difference in STH-infected subjects between albendazole and ivermectin plus albendazole, compared to a slightly higher incidence of AEs in filariasis patients treated with ivermectin plus albendazole, which appears to be mostly related to Mazzotti-type reactions (*i*.*e*. caused by the effects of treatment of microfilariae).

A common problem when assessing safety in clinical trials is that they tend to report the incidence of AEs on treatment disregarding their presence and intensity before treatment. Here, we identified eight studies which took into account pre-treatment symptoms and clinical parameters; among these only three (all in filariasis) showed increased numbers of AEs compared to the baseline status in co-treated patients [40, 49, 56].

The main limitation of this systematic review is the very low number of published RCTs on the efficacy (n = 4) and safety (n = 5) of ivermectin-albendazole compared to albendazole or ivermectin alone, which meant we could only conduct a meta-analysis on *T*. *trichiura* infections, but not on the other two STH species. Sub-group analysis (e.g. by population strata, level of baseline worm burden, parasitic disease for safety data) was also either not possible or inconclusive with such low numbers of studies. While our meta-analysis focused on the risk of still being infected after treatment, a future analysis, once more data from upcoming studies will be available, could consider using mixed linear models for analyzing egg reduction rates, a key parameter for assessing anthelminthic drug efficacy. The low number of eligible studies also prevented us from evaluating the possibility of publication bias [27] or heterogeneity for certain sub-groups. Moreover, the included studies revealed several shortcomings. Of the four studies selected to summarize overall efficacy, two [20, 24] presented low risk of bias as per the Cochrane risk of bias tool but the remaining two studies [18, 19] were not double-blinded and did not report on several procedures. Moreover, two of the included studies [18, 20] did not adhere to the recommended follow up time point 2–3 weeks post-treatment [66]. Finally, within the five safety-related studies eligible for the meta-analysis, three reached acceptable quality levels with at least five out of the six bias indicators considered as “low risk”.

It is therefore obvious that our findings need to be confirmed through high-quality research studies with a rigorous design (e.g. single- or double-blinded RCTs). Furthermore, it is important to produce evidence from different settings in terms of parasite and target populations. The main STH species plus *S*. *stercoralis* may show different drug susceptibility in different endemic areas potentially also related to different levels of drug pressure in populations to be treated (e.g. MDA naïve *vs*. experienced populations) [67, 68]. These studies could also evaluate the long-term benefit and different treatment schedules of this co-administration, compare ivermectin-albendazole to other co-administration treatments, which have emerged over the past years [69], to inform treatment guidelines and strategic planning of STH control, as well as monitoring and evaluation. Along with efficacy, future studies must assess safety in the same rigorous manner. The safety review suggests that AEs of ivermectin-albendazole may be more common in populations with filariasis. Since filariasis and soil-transmitted helminthiases often co-exist [70], future studies must take into account filariasis either by excluding co-endemic areas or by diagnosing both diseases.

### Conclusions

Our results suggest that the co-administration of ivermectin and albendazole increases efficacy against *T*. *trichiura*, but most likely has no gain against *A*. *lumbricoides* and hookworm. Safety reports were very diverse in study design, target population and treatment indication but in summary confirmed its tolerability with mostly mild and transient AEs. Together, these findings support the recent WHO recommendations and inclusion in the WHO Model List of Essential Medicines. At the same time, they also point to the need for additional and more reliable information through well-conducted studies in the different contexts where the co-administration is to be deployed. A safety-related shortcoming of the ivermectin-albendazole co-administration is that it cannot be deployed in areas where also *Loa loa* is prevalent, since ivermectin is known to produce severe and possibly fatal adverse reactions such as neurological signs, encephalopathy and coma in heavily infected individuals and is thus contraindicated in endemic West and Central Africa [9]. Alternative treatments with excellent trichuricidal activity are therefore required. Oxantel pamoate might fill this gap. The drug has been thoroughly studied over the past years and in combination with albendazole has shown a high broad spectrum activity against all STH [24, 71–73] and hence might serve as an excellent alternative to albendazole-ivermectin.

## Supporting information

S1 File Review protocol used. (PDF)

S2 File PROSPERO protocol registration CRD42017060710. (PDF)

S3 File PRISMA checklist. (PDF)

S1 Text Search strategy applied for review on efficacy and safety of ivermectin-albendazole co-administration. (DOCX)

S2 Text Tables listing main characteristics of potentially relevant studies reporting efficacy data against STH infections (A) or safety data (B) on ivermectin-albendazole co-administration.
(DOCX)
