# Supplementary material for: Efficacy and safety of co-administered ivermectin plus albendazole for treating soil-transmitted helminths: A systematic review, meta-analysis and individual patient data analysis
Source: PLoS Negl Trop Dis. 2018 Apr 27;12(4):e0006458. doi: 10.1371/journal.pntd.0006458 (PMC5942849; doi:10.1371/journal.pntd.0006458)
Supplement: S1 File — (PDF) [file pntd.0006458.s001.pdf]

Efficacy and safety of co-administered ivermectin plus albendazole for treating soil-transmitted helminths: a systematic review meta-analysis and individual patient data analysis

Review protocol

Organization, City, Country: Swiss Tropical and Public Health Institute, Basel, Switzerland

Prepared by: Eveline Hürlimann and Marta Palmeirim

Date: 01.05.2017

Senior supervisor: Jennifer Keiser

Research team: Eveline Hürlimann, Marta Palmeirim, Serene Joseph, Michel Vaillant, Piero Olliaro, Jennifer Keiser

Table of Contents

1. Background 3

2. Objective 4

3. Review question 4

4. Protocol registration 5

5. Evidence gathering and study selection 5

5.1. Evidence gathering 5

5.1.1. Searching databases 5

5.1.2. Hand searching 5

5.1.3. Reference searches 5

5.2. Eligibility criteria 6

6. Assessment of risk of bias and data extraction 6

7. References 7

1. Background

Parasitic worms, particularly soil-transmitted helminths (STHs), infect over a billion of the world’s population and they are the most common infectious in low- and middle-income countries (LMICs) [1, 2]. Helminthiases cause considerable burden, including physical and intellectual growth retardation among preschool- and school-aged children [3-5]. Despite their negative impact on public health, education, social and economic development, helminthiases are still often neglected. Helminthiases account for the largest burden of the neglected tropical diseases (NTDs) [6-8]. They are most prevalent in LMICs, particularly in sub-Saharan Africa, East Asia, Southeast Asia and South America, governed by inadequate water supply and sanitation, crowded living conditions, difficult access to health care and low levels of education [9, 10].

STHs infect humans either through faecal-oral route or through contact with soil contaminated with parasite eggs or larvae that thrive in warm and moist soils, which explains why they are most common in tropical and sub-tropical regions. The soil becomes contaminated when an infected individual defecates helminth eggs into the environment. These eggs are extremely resistant and may persist in the soil for several years until they find a host to infect [11]. Most helminthiasis are caused by *Ascaris lumbricoides*, *Trichuris trichiura* and hookworm. In the case of *A. lumbricoides* and *T. trichiura*, the infection takes place through oral ingestion of mature worm eggs. Eggs can be found on fruit or vegetables which were not properly washed, untreated drinking water or dirty hands, for example. In the case of hookworm (*Ancylostoma duodenale* and *Necator americanus*), eggs are also passed to the environment through an infected host’s stool where, under favourable conditions, they hatch and release larvae which can actively penetrate the skin to infect new individuals [12].

Currently, STH infections are treated predominantly with albendazole (400 mg) or mebendazole (500 mg) which are usually distributed in large-scale programmes to children and women of childbearing age [2, 13]. Albendazole and mebendazole are recommended due to their high safety profile and ease of administration (*i.e.* single-dose drugs that can be administered once or twice per year). A meta-analysis by Keiser and Utzinger has shown that these two drugs are highly efficacious against *A. lumbricoides* (cure rate, CR = 88% with albendazole and CR = 95% with mebendazole) and moderately efficacious against hookworm (CR = 72% with albendazole and CR = 15% with mebendazole). However, the same meta-analysis found unsatisfactory overall CRs of these drugs against *T. trichiura* (CR = 28% with albendazole and CR = 36% with mebendazole) [14]. Therefore, it is crucial to increase efforts towards finding alternative therapies to both increase CRs and delay the insurgence of drug resistance. There are different approaches to finding alternative treatments. One approach is to develop new drugs, however the drug discovery pipeline is

empty. Hence, there is a need to evaluate and implement innovative strategies, including drug combinations.

Ivermectin has been used extensively, alone against onchocerciasis and in combination with albendazole against lymphatic filariasis since the 1980s [15]. This drug has played a key role in the elimination programmes of these two NTDs. The co-administration of ivermectin and albendazole is already recommended in areas where STH infections are co-endemic with lymphatic filariasis and/or onchocerciasis. The overlapping geographical distribution of these diseases and of large-scale control programmes has led to evidence on the positive impact of ivermectin on STH infections [16]. Given the trichuricidal properties of ivermectin, adding this drug to albendazole would broaden the efficacy of the anthelminthic treatment. Albendazole-ivermectin is the frontrunner of anthelminthic drug combinations, given that both drugs are marketed and have been widely used against lymphatic filariasis.

2. Objective

This review aims at comparing the efficacy and safety of the co-administration of ivermectin and albendazole to the administration of each of these drugs alone against STH which could lead to an increase of evidence to improve the currently recommended control strategies. To our knowledge, this is the first systematic review including a meta-analysis comparing these two therapies.

3. Review question

For the purposes of this literature review, the population, intervention, comparators and outcomes (PICO) framework to inform the review objectives are presented below.

Population	Intervention	Comparison	Outcome
People in countries where STH infections are endemic	Co-administration of ivermectin and albendazole	Administration of ivermectin and albendazole alone	**Efficacy**: number of infected, number of cured, cure rate (CR) and egg reduction rate (ERR); **Safety**: number and type of AEs and specific reported symptoms (when available).

4. Protocol registration

This review protocol will be recorded and published in the International Prospective Register of Systematic Reviews (PROSPERO) online database. This study will be reported in accordance with PRISMA guidelines.

5. Evidence gathering and study selection

5.1. Evidence gathering

The evidence gathering approach will have three components:

5.1.1. Searching databases

The databases listed below will be searched with a pre-determined strategy as detailed in Appendix 1. In cases where the search results are small in number, search terms will be reduced to maximize the search sensitivity.

- PubMed
- ISI Web of Science
- Science Direct

5.1.2. Hand searching

The following websites will be hand-searched for relevant articles:

- Cochrane Central Registration of Clinical Trials
- www.clinicaltrials.gov

5.1.3. Reference searches

Bibliographies of those papers that match the eligibility criteria below will be searched by hand to identify any further, relevant references, which will undergo the same screening and selection process.

5.2. Eligibility criteria

After the gathering of evidence, all identified references will be screened independently by two reviewers (Eveline Hürlimann and Marta Palmeirim) using a three-stage approach to reviewing the title, abstract and full text. The following eligibility criteria will be applied:

Efficacy studies

Only randomized controlled trials which tested ivermectin and albendazole against at least one STH (hookworm, *T. trichiura* and/or *A. lumbricoides*), which administered the recommended doses of ivermectin and/or albendazole (ivermectin: 200ug/kg; albendazole: 400mg) and which assessed the drug efficacy (follow-up survey) between 7 days and six weeks post-treatment were eligible for inclusion. Additionally, only studies which provide number of treated and cured for each treatment arm (or number of treated and CR) will be included. Reviews will be regarded as irrelevant if they do not provide any additional original data that is not yet published elsewhere.

Safety studies

Any study reporting quantitative or qualitative data of adverse events from administration of ivermectin in combination with albendazole. Case studies from medical reports will not be considered for inclusion due to non-representativeness of outcomes. Otherwise all studies providing population-based (e.g. community members, schoolchildren or hospital/diagnosed patients) data will be included even when the study sample size is relatively low. Reviews will be regarded as irrelevant if they do not provide any additional original data that is not yet published elsewhere.

6. Assessment of risk of bias and data extraction

Data extraction from relevant papers will take place using predefined summary templates attached in Appendix 2 (efficacy) and Appendix 3 (safety). All data will be collected regarding the eligibility criteria.

The quality and risk of bias of eligible efficacy studies will be done at study level using the Cochrane risk of bias tool [17]. If sufficient studies are identified, risk of bias across studies will be assessed using a funnel plot.

7. References

1. Pullan, R.L., J.L. Smith, R. Jasrasaria, and S.J. Brooker, *Global numbers of infection and disease burden of soil transmitted helminth infections in 2010.* Parasit Vectors, 2014. **7**: p. 37.
2. Hotez, P.J., D.H. Molyneux, A. Fenwick, J. Kumaresan, S.E. Sachs, J.D. Sachs, and L. Savioli, *Control of neglected tropical diseases.* N Engl J Med, 2007. **357**(10): p. 1018-27.
3. Anderson, R.M., J.E. Truscott, R.L. Pullan, S.J. Brooker, and T.D. Hollingsworth, *How effective is school-based deworming for the community-wide control of soil-transmitted helminths?* PLoS Negl Trop Dis, 2013. **7**(2): p. e2027.
4. Tomono, N., M.T. Anantaphruti, P. Jongsuksuntigul, P. Thongthien, P. Leerapan, Y. Silapharatsamee, S. Kojima, and S. Looareesuwan, *Risk factors of helminthiases among schoolchildren in southern Thailand.* Southeast Asian J Trop Med Public Health, 2003. **34**(2): p. 264-8.
5. Bethony, J., S. Brooker, M. Albonico, S.M. Geiger, A. Loukas, D. Diemert, and P.J. Hotez, *Soil-transmitted helminth infections: ascariasis, trichuriasis, and hookworm.* Lancet, 2006. **367**(9521): p. 1521-32.
6. Hotez, P.J., M. Alvarado, M.G. Basáñez, I. Bolliger, R. Bourne, M. Boussinesq, S.J. Brooker, A.S. Brown, G. Buckle, C.M. Budke, H. Carabin, L.E. Coffeng, E.M. Fevre, T. Furst, Y.A. Halasa, R. Jasrasaria, N.E. Johns, J. Keiser, C.H. King, R. Lozano, M.E. Murdoch, S. O'Hanlon, S.D. Pion, R.L. Pullan, K.D. Ramaiah, T. Roberts, D.S. Shepard, J.L. Smith, W.A. Stolk, E.A. Undurraga, J. Utzinger, M. Wang, C.J. Murray, and M. Naghavi, *The global burden of disease study 2010: interpretation and implications for the neglected tropical diseases.* PLoS Negl Trop Dis, 2014. **8**(7): p. e2865.
7. Utzinger, J., S.L. Becker, S. Knopp, J. Blum, A.L. Neumayr, J. Keiser, and C.F. Hatz, *Neglected tropical diseases: diagnosis, clinical management, treatment and control.* Swiss Med Wkly, 2012. **142**: p. w13727.
8. Murray, C.J.L., *Quantifying the burden of disease: the technical basis for disability-adjusted life years.* Bull World Health Organ, 1994. **72**(3): p. 429-45.
9. Alum, A., J.R. Rubino, and M.K. Ijaz, *The global war against intestinal parasites--should we use a holistic approach?* Int J Infect Dis, 2010. **14**(9): p. e732-8.
10. Mascarini-Serra, L., *Prevention of Soil-transmitted Helminth Infection.* J Glob Infect Dis, 2011. **3**(2): p. 175-182.
11. Addiss, D., *Tackling worms in children: school programmes can work - for eyes too.* Community Eye Health, 2013. **26**(82): p. 29-31.

12. Cross, J.H., *Enteric nematodes of humans*, in *Medical Microbiology*. 1996, University of Texas Medical Branch Galveston, Texas, USA.
13. WHO, *Preventive chemotherapy in human helminthiasis - Coordinated use of anthelminthic drugs in control interventions: a manual for health professionals and programme managers*. 2006, Geneva: World Health Organization. 62.
14. Keiser, J. and J. Utzinger, *Efficacy of current drugs against soil-transmitted helminth infections: systematic review and meta-analysis.* JAMA, 2008. **299**(16): p. 1937-48.
15. WHO, *Helminth control in school-age children: a guide for managers of control programmes.* 2011.
16. Krotneva, S.P., L.E. Coffeng, M. Noma, H.G. Zoure, L. Bakone, U.V. Amazigo, S.J. de Vlas, and W.A. Stolk, *African Program for Onchocerciasis Control 1995-2010: Impact of Annual Ivermectin Mass Treatment on Off-Target Infectious Diseases.* PLoS Negl Trop Dis, 2015. **9**(9): p. e0004051.
17. Higgins, J.P., D.G. Altman, P.C. Gotzsche, P. Juni, D. Moher, A.D. Oxman, J. Savovic, K.F. Schulz, L. Weeks, and J.A. Sterne, *The Cochrane Collaboration's tool for assessing risk of bias in randomised trials.* Bmj, 2011. **343**: p. d5928.

Appendix 1. Pre-determined search strategy

Efficacy studies		
**Database**	**Key words**	**Filters**
PubMed	(ivermect* AND albendaz* AND (hookworm OR trichuri* OR ascari* OR soil-transmitted helminth*) AND (cure* OR trial))	No filter
ISI Web of Science		No filter
Science Direct		Excluded books
Cochrane Central Registration of CTs	Ivermectin AND albendazole	No filter
WHO Library Database		No filter
Clinicaltrials.gov		No filter

Safety studies		
**Database**	**Key words**	**Filters**
PubMed	(ivermect* [AND] alben* [AND] combin* [AND] (adverse [OR] side effect* [OR] symptom*))	No filter
ISI Web of Science		No filter
Science Direct		Excluded books
Cochrane Central Registration of CTs	Ivermectin AND albendazole	No filter
WHO Library Database		No filter
Clinicaltrials.gov		No filter

Appendix 2. Data entry templates – efficacy

study	
comb_total_tri	
comb_cured_tri	
comb_not_tri	
cr_comb_tri	
comb_total_asc	
comb_cured_asc	
comb_not_asc	
cr_comb_asc	
comb_total_ank	
comb_cured_ank	
comb_not_ank	
cr_comb_ank	
err_comb_tri	
err_comb_asc	
err_comb_ank	
alb_total_tri	
alb_cured_tri	
alb_not_tri	
cr_alb_tri	
alb_total_asc	
alb_cured_asc	
alb_not_asc	
cr_alb_asc	
alb_total_ank	
alb_cured_ank	
alb_not_ank	
cr_alb_ank	
err_alb_tri	
err_alb_asc	
err_alb_ank	
ivm_total_tri	
ivm_cured_tri	
cr_ivm_tri	
ivm_total_asc	
ivm_cured_asc	
cr_ivm_asc	
ivm_total_ank	
ivm_cured_ank	
cr_ivm_ank	
err_ivm_tri	
err_ivm_asc	
err_ivm_ank	

Appendix 3. Data entry templates – safety

study_nb	
author year	
parasite	
comb_aes	
comb_not_aes	
comb_total	
ivm_aes	
ivm_not_aes	
ivm_total	
alb_aes	
alb_not_aes	
alb_total	
