# Supplementary material for: Efficacy and safety of co-administered ivermectin plus albendazole for treating soil-transmitted helminths: A systematic review, meta-analysis and individual patient data analysis
Source: PLoS Negl Trop Dis. 2018 Apr 27;12(4):e0006458. doi: 10.1371/journal.pntd.0006458 (PMC5942849; doi:10.1371/journal.pntd.0006458)
Supplement: S2 File — (PDF) [file pntd.0006458.s002.pdf]

Systematic review

1. * Review title.

Give the working title of the review, for example the one used for obtaining funding. Ideally the title should state succinctly the interventions or exposures being reviewed and the associated health or social problems. Where appropriate, the title should use the PI(E)COS structure to contain information on the Participants, Intervention (or Exposure) and Comparison groups, the Outcomes to be measured and Study designs to be included.
Efficacy and safety of co-administration of ivermectin and albendazole for the treatment of soil-transmitted helminths: a systematic review and meta-analysis

2. Original language title.

For reviews in languages other than English, this field should be used to enter the title in the language of the review. This will be displayed together with the English language title.

3. * Anticipated or actual start date.

Give the date when the systematic review commenced, or is expected to commence.
01/11/2016

4. * Anticipated completion date.

Give the date by which the review is expected to be completed.
30/11/2017

5. * Stage of review at time of this submission.

Indicate the stage of progress of the review by ticking the relevant Started and Completed boxes. Additional information may be added in the free text box provided.
Please note: Reviews that have progressed beyond the point of completing data extraction at the time of initial registration are not eligible for inclusion in PROSPERO. Should evidence of incorrect status and/or completion date being supplied at the time of submission come to light, the content of the PROSPERO record will be removed leaving only the title and named contact details and a statement that inaccuracies in the stage of the review date had been identified.
This field should be updated when any amendments are made to a published record and on completion and publication of the review.

The review has not yet started: No

**Review stage**	**Started**	**Completed**
Preliminary searches	Yes	Yes
Piloting of the study selection process	Yes	Yes
Formal screening of search results against eligibility criteria	Yes	Yes
Data extraction	Yes	Yes
Risk of bias (quality) assessment	Yes	Yes
Data analysis	Yes	No

Provide any other relevant information about the stage of the review here (e.g. Funded proposal, protocol not

yet finalised).
Data analysis ongoing, publication to be finalised

Data analysis ongoing, publication to be finalised

6. * Named contact.

The named contact acts as the guarantor for the accuracy of the information presented in the register record.
Eveline Hürlimann

Email salutation (e.g. "Dr Smith" or "Joanne") for correspondence:

7. * Named contact email.

Give the electronic mail address of the named contact.
eveline.huerlimann@swisstph.ch

8. Named contact address

Give the full postal address for the named contact.
Swiss Tropical and Public Health Institute (Swiss TPH)
Department of Medical Parasitology and Infection Biology
Socinstrasse 57
P.O. Box
CH-4002 Basel
Switzerland

9. Named contact phone number.

Give the telephone number for the named contact, including international dialling code.
+41612848704

10. * Organisational affiliation of the review.

Full title of the organisational affiliations for this review and website address if available. This field may be completed as 'None' if the review is not affiliated to any organisation.
Swiss Tropical Public Health Institute, University of Basel

Organisation web address:

https://www.swisstph.ch/en/

11. Review team members and their organisational affiliations.

Give the title, first name, last name and the organisational affiliations of each member of the review team. Affiliation refers to groups or organisations to which review team members belong.

Dr Eveline Hürlimann. Swiss Tropical and Public Health Institute
Ms Marta Palmeirim. Swiss Tropical and Public Health Institute
Dr Jennifer Keiser. Swiss Tropical and Public Health Institute
Dr Serene Joseph. Swiss Tropical and Public Health Institute

12. * Funding sources/sponsors.

Give details of the individuals, organizations, groups or other legal entities who take responsibility for initiating, managing, sponsoring and/or financing the review. Include any unique identification numbers assigned to the review by the individuals or bodies listed.
Bill and Melinda Gates Foundation

13. * Conflicts of interest.

List any conditions that could lead to actual or perceived undue influence on judgements concerning the main topic investigated in the review.

None

14. Collaborators.

Give the name and affiliation of any individuals or organisations who are working on the review but who are not listed as review team members.

Dr Piero Olliaro. WHO TDR, Geneva, Switzerland
Dr Michel Vaillant. Luxembourg Institute of Health
Dr Antonio Montresor. WHO, Geneva, Switzerland

15. * Review question.

State the question(s) to be addressed by the review, clearly and precisely. Review questions may be specific or broad. It may be appropriate to break very broad questions down into a series of related more specific questions. Questions may be framed or refined using PI(E)COS where relevant.
What is the current evidence regarding the efficacy of co-administered ivermectin and albendazole against soil-transmitted helminths?
Is co-administered ivermectin and albendazole more effective against soil-transmitted helminth infections than single drug regimens (albendazole or ivermectin alone)?
What is known about safety parameters for the co-administration of ivermectin and albendazole, and are they different to those for single drug administration?

16. * Searches.

Give details of the sources to be searched, search dates (from and to), and any restrictions (e.g. language or publication period). The full search strategy is not required, but may be supplied as a link or attachment.
With regard to the efficacy of co-administration of ivermectin and albendazole, a literature search was performed in PubMed, with no restrictions on language (from 1960 to November 2016), to identify clinical trials, studies and case reports pertaining to the use of ivermectin for treating hookworm, A. lumbricoides and T. trichiura.
The search criteria was: “ivermect* AND (hookworm OR trichuri* OR ascari* OR soil-transmitted helminth*) AND (cure* OR trial)”.
To identify safety data on co-administered ivermectin and albendazole, a second literature search using PubMed (from 1960 to November 2016) was performed using the following search criteria: "ivermect* AND alben* AND combin* AND (adverse OR side effect* OR symptom*)". No restrictions on language of publication, parasite species treated or study type were applied.
The search results can be viewed at: https://www.ncbi.nlm.nih.gov/pubmed?term=((ivermect*%20AND%20(hookworm%20OR%20trichuri*%20OR%20ascari*%20OR%20soil-transmitted%20helminth*)%20AND%20(cure*%20OR%20trial)))%20AND%20(%221960%22%5BDate%20-%20Publication%5D%20%3A%20%222016%2F11%2F01%22%5BDate%20-%20Publication%5D) and https://www.ncbi.nlm.nih.gov/pubmed/?term=((((((((((ivermect*)+AND+alben*))+AND+combin*)))+AND+(adverse+OR+side+effect*+OR+symptom*)))))+AND+(%221960%22%5BDate+-+Publication%5D+%3A+%222016%2F11%2F01%22%5BDate+-+Publication%5D)

17. URL to search strategy.

Give a link to the search strategy or an example of a search strategy for a specific database if available (including the keywords that will be used in the search strategies).

https://www.crd.york.ac.uk/PROSPEROFILES/60710_STRATEGY_20171110.pdf

Alternatively, upload your search strategy to CRD in pdf format. Please note that by doing so you are consenting to the file being made publicly accessible.

Yes I give permission for this file to be made publicly available

18. * Condition or domain being studied.

Give a short description of the disease, condition or healthcare domain being studied. This could include health and wellbeing outcomes.
Soil-transmitted helminthiases (STH), including infection with Ascaris lumbricoides, Trichuris trichiura, or the two hookworm species Necator americanus and/or Ankylostoma duodenale, as determined by egg output in the stool.

19. * Participants/population.

Give summary criteria for the participants or populations being studied by the review. The preferred format includes details of both inclusion and exclusion criteria.
Given that there are very few studies on the co-administration of albendazole and ivermectin against soil-transmitted helminths, in order to ensure as comprehensive a coverage of the available evidence as possible, no studies were excluded based on population.
Inclusion (efficacy): STH-infected individuals treated with ivermectin alone or in combination with albendazole in randomized-controlled trials.
Inclusion (safety): any population regardless of specific infection status treated with ivermectin and albendazole in combination.
Exclusion: case studies.

20. * Intervention(s), exposure(s).

Give full and clear descriptions or definitions of the nature of the interventions or the exposures to be reviewed.
From the studies retrieved from the literature search regarding efficacy, we will select studies with a randomized controlled trial (RCT) design which have tested the effects of the following therapies against at least one STH (hookworm, T. trichiura and/or A. lumbricoides): (1) co-administration of ivermectin and albendazole; (2) ivermectin alone (or with placebo).
Efficacy outcomes may include cure rates (CRs) and egg reduction rates (ERRs) but will need to provide as a minimal level of information the total number of participants and the number of positives or no-longer infected, respectively, at baseline and follow-up. Only studies which have administered the recommended doses of ivermectin and/or albendazole (ivermectin: 200ug/kg; albendazole: 400mg) and which have assessed the drug efficacy (follow-up survey) between two and six weeks post-treatment will be included.
With regard to safety data, all studies reporting any quantitative information on adverse events (AEs) or symptoms assessed pre- and post-treatment after co-administration of ivermectin and albendazole will be considered. This may include information on severity of AEs, frequencies of AEs and symptoms for co-administered ivermectin and albendazole and if available from albendazole alone as comparator. Factors (patient, health-system or baseline infection status-related) influencing safety will be discussed in a more qualitative way. Studies will be included irrespective of the indication (parasite species) and dose regimen, and information from reviews may be considered unless it has already been published elsewhere. Case reports will be excluded.

21. * Comparator(s)/control.

Where relevant, give details of the alternatives against which the main subject/topic of the review will be compared (e.g. another intervention or a non-exposed control group). The preferred format includes details of both inclusion and exclusion criteria.
We intend to compare the efficacy and safety of co-administered ivermectin and albendazole to the single drug regimens (albendazole or ivermectin alone).

22. * Types of study to be included.

Give details of the types of study (study designs) eligible for inclusion in the review. If there are no restrictions on the types of study design eligible for inclusion, or certain study types are excluded, this should be stated. The preferred format includes details of both inclusion and exclusion criteria.
For the review on efficacy, only randomized controlled trials will be considered. Clinical trials which have not been randomized, or which have no control group, will be excluded.Safety references may stem from any

type of study, except from case studies, which will be considered irrelevant.

23. Context.

Give summary details of the setting and other relevant characteristics which help define the inclusion or exclusion criteria.
No context criteria applied.

24. * Primary outcome(s).

Give the pre-specified primary (most important) outcomes of the review, including details of how the outcome is defined and measured and when these measurement are made, if these are part of the review inclusion criteria.
Efficacy of co-administered ivermectin and albendazole compared to single drug treatment (albendazole or ivermectin alone) against any of the three soil-transmitted helminths (A. lumbricoides, T. trichiura or/and hookworm) as assessed through CRs and ERRs within studies and RRs between studies.

Timing and effect measures

Effect measures: cure rates (CRs), egg reduction rates (ERRs), relative risk (RR) in meta-analysis.
Timing: post-treatment evaluation period of between 2 and 6 weeks.

25. * Secondary outcome(s).

List the pre-specified secondary (additional) outcomes of the review, with a similar level of detail to that required for primary outcomes. Where there are no secondary outcomes please state ‘None’ or ‘Not applicable’ as appropriate to the review
In-depth analysis of results on efficacy based on obtained individual patient data.
Safety of ivermectin and albendazole co-adminstration, in terms of number of patients with AEs, compared to single drug regimens (albendazole or ivermectin alone).
Frequency and types of adverse events resulting from the co-administration of ivermectin and albendazole.
Qualitative summary on factors additionally influencing safety of co-administered ivermectin and albendazole.

Timing and effect measures

Effect measures: relative risk (RR) in meta-analysis.

26. Data extraction (selection and coding).

Give the procedure for selecting studies for the review and extracting data, including the number of researchers involved and how discrepancies will be resolved. List the data to be extracted.
The titles/abstracts of the studies retrieved from the literature search of PubMed, and stored in EndNote, will be screened for eligibility, as outlined above, by two review team members. Any disagreements between the reviewers, or uncertainties regarding the relevance of a particular study for inclusion, will be resolved through discussion with a third reviewer. In addition, to facilitate later data extraction and classification of data quality, the reviewers will highlight relevant text sections in the retrieved PDFs, which will then be attached to the EndNote reference, together with a number of key words for labelling the respective publications with respect to content and the inclusion or exclusion criteria.
A pre-piloted Excel form will be used to extract data from the included studies for evidence synthesis. For each study, information on the publication (i.e., authors and year), general study-specific data such as type of study, country where the study took place, parasite species, participant data (i.e., age group, number of individuals), follow-up period and data collection method (i.e., repeated stool sampling for efficacy, passive vs. active surveillance for safety) will be retrieved.
For studies assessing efficacy, the main outcomes to be extracted will consist of the number of treated and infected participants (before and after treatment), the cure rates (CRs) and the egg reduction rates (ERRs) (if available).
The number of AEs and specific reported symptoms (if detailed), the types of AEs (i.e., their symptoms, and whether they were observable or laboratory events) and whether the AEs were associated with baseline

parasite infection status will be recorded for the appraisal of the safety data.
Patient individual data from relevant publications will be gained through personal communication with the study authors.

27. * Risk of bias (quality) assessment.

State whether and how risk of bias will be assessed (including the number of researchers involved and how discrepancies will be resolved), how the quality of individual studies will be assessed, and whether and how this will influence the planned synthesis.
The quality and risk of bias of eligible efficacy studies will be done at study level using the Cochrane risk of bias tool. If sufficient studies are identified, risk of bias across studies will be assessed using a funnel plot.

28. * Strategy for data synthesis.

Give the planned general approach to synthesis, e.g. whether aggregate or individual participant data will be used and whether a quantitative or narrative (descriptive) synthesis is planned. It is acceptable to state that a quantitative synthesis will be used if the included studies are sufficiently homogenous.
Individual patient data analysis.
Efficacy for studies providing results for the combined ivermectin-albendazole treatment as well as for the single drug regimen (i.e., albendazole alone or ivermectin alone) as a comparator will be used for a meta-analysis. Likewise, safety data from studies with a combination, and a single regimen treatment arm (i.e. albendazole or ivermectin alone) and providing the minimum quantitative data necessary, will be subjected to meta-analysis.
General information relating to the current evidence on the efficacy and safety of the co-administration of ivermectin and albendazole will be summarized with descriptive statistics, and discussed in a narrative approach.

29. * Analysis of subgroups or subsets.

Give details of any plans for the separate presentation, exploration or analysis of different types of participants (e.g. by age, disease status, ethnicity, socioeconomic status, presence or absence or co-morbidities); different types of intervention (e.g. drug dose, presence or absence of particular components of intervention); different settings (e.g. country, acute or primary care sector, professional or family care); or different types of study (e.g. randomised or non-randomised).
None planned for efficacy, if enough studies qualify for meta-analysis for safety, a sub-group analysis for type of parasitic disease (e.g. STH, onchocerciasis, LF) will be done.

30. * Type and method of review.

Select the type of review and the review method from the lists below. Select the health area(s) of interest for your review.

Type of review

Cost effectiveness
No

Diagnostic
No

Epidemiologic
No

Individual patient data (IPD) meta-analysis
No

Intervention
No

Meta-analysis
Yes

Methodology
No

Network meta-analysis
No

Pre-clinical
No

Prevention
No

Prognostic
No

Prospective meta-analysis (PMA)
No

Qualitative synthesis
No

Review of reviews
No

Service delivery
No

Systematic review
Yes

Other
No

Health area of the review

Alcohol/substance misuse/abuse
No

Blood and immune system
No

Cancer
No

Cardiovascular
No

Care of the elderly
No

Child health
No

Complementary therapies
No

Crime and justice
No

Dental
No

Digestive system
No

Ear, nose and throat
No

Education
No

Endocrine and metabolic disorders
No

Eye disorders
No

General interest
No

Genetics
No

Health inequalities/health equity
No
Infections and infestations
Yes
International development
No
Mental health and behavioural conditions
No
Musculoskeletal
No
Neurological
No
Nursing
No
Obstetrics and gynaecology
No
Oral health
No
Palliative care
No
Perioperative care
No
Physiotherapy
No
Pregnancy and childbirth
No
Public health (including social determinants of health)
Yes
Rehabilitation
No
Respiratory disorders
No
Service delivery
No
Skin disorders
No
Social care
No
Tropical Medicine
Yes
Urological
No
Wounds, injuries and accidents
No
Violence and abuse
No

31. Language.

Select each language individually to add it to the list below, use the bin icon to remove any added in error.
English

There is an English language summary.

32. Country.

Select the country in which the review is being carried out from the drop down list. For multi-national collaborations select all the countries involved.

Switzerland

33. Other registration details.

Give the name of any organisation where the systematic review title or protocol is registered (such as with The Campbell Collaboration, or The Joanna Briggs Institute) together with any unique identification number assigned. (N.B. Registration details for Cochrane protocols will be automatically entered). If extracted data will be stored and made available through a repository such as the Systematic Review Data Repository (SRDR), details and a link should be included here. If none, leave blank.

34. Reference and/or URL for published protocol.

Give the citation and link for the published protocol, if there is one

Give the link to the published protocol.

Alternatively, upload your published protocol to CRD in pdf format. Please note that by doing so you are consenting to the file being made publicly accessible.

Yes I give permission for this file to be made publicly available

Please note that the information required in the PROSPERO registration form must be completed in full even if access to a protocol is given.

35. Dissemination plans.

Give brief details of plans for communicating essential messages from the review to the appropriate audiences.

Do you intend to publish the review on completion?

Yes

36. Keywords.

Give words or phrases that best describe the review. Separate keywords with a semicolon or new line. Keywords will help users find the review in the Register (the words do not appear in the public record but are included in searches). Be as specific and precise as possible. Avoid acronyms and abbreviations unless these are in wide use.

Soil-transmitted helminths
Efficacy
Safety
Ivermectin
Albendazole
Co-administration

37. Details of any existing review of the same topic by the same authors.

Give details of earlier versions of the systematic review if an update of an existing review is being registered, including full bibliographic reference if possible.

38. * Current review status.

Review status should be updated when the review is completed and when it is published.

Please provide anticipated publication date

Review_Completed_not_published

39. Any additional information.

Provide any other information the review team feel is relevant to the registration of the review.

40. Details of final report/publication(s).

This field should be left empty until details of the completed review are available.

Give the link to the published review.
