# Supplementary material for: Efficacy and safety of co-administered ivermectin plus albendazole for treating soil-transmitted helminths: A systematic review, meta-analysis and individual patient data analysis
Source: PLoS Negl Trop Dis. 2018 Apr 27;12(4):e0006458. doi: 10.1371/journal.pntd.0006458 (PMC5942849; doi:10.1371/journal.pntd.0006458)
Supplement: S1 Text — (DOCX) [file pntd.0006458.s004.docx]

SEARCH STRATEGY

Ivermectin + albendazole review

Efficacy studies

| **Database** | **Key words** | **Filters** | **Hits** | **New hits** | **New relevant studies** | **Date** |
| --- | --- | --- | --- | --- | --- | --- |
| PubMed | (ivermect* AND albendaz* AND (hookworm OR trichuri* OR ascari* OR soil-transmitted helminth*) AND (cure* OR trial)) | No filter | 21 | 21 | 7 | 24.01.18 |
| ISI Web of Science |  | No filter | 47 | 29 | 0 | 24.01.18 |
| Science Direct |  | Excluded books | 431 | 396 | 0 | 24.01.18 |
| Cochrane Central Registration of CTs | Ivermectin AND albendazole | No filter | 77 | 55 | 0 | 24.01.18 |
| Clinicaltrials.gov |  | No filter | 31 | 23 | 0 | 24.01.18 |

Safety studies

| **Database** | **Key words** | **Filters** | **Hits** | **New hits** | **New relevant studies** | **Date** |
| --- | --- | --- | --- | --- | --- | --- |
| PubMed | (ivermect* [AND] alben* [AND] combin* [AND] (adverse [OR] side effect* [OR] symptom*)) | No filter | 71 | 71 | 23 | 24.01.18 |
| ISI Web of Science |  | No filter | 59 | 24 | 1 | 24.01.18 |
| Science Direct |  | Excluded books | 522 | 503 | 3 | 24.01.18 |
| Cochrane Central Registration of CTs | Ivermectin AND albendazole | No filter | 77 | 44 | 4 | 24.01.18 |
| Clinicaltrials.gov |  | No filter | 31 | 23 | 1 | 24.01.18 |
