# Supplementary material for: Efficacy and safety of co-administered ivermectin plus albendazole for treating soil-transmitted helminths: A systematic review, meta-analysis and individual patient data analysis
Source: PLoS Negl Trop Dis. 2018 Apr 27;12(4):e0006458. doi: 10.1371/journal.pntd.0006458 (PMC5942849; doi:10.1371/journal.pntd.0006458)
Supplement: S2 Text — Tables listing main characteristics of potentially relevant studies reporting efficacy data against STH infections (A) or safety data (B) on ivermectin-albendazole co-administration. (DOCX) [file pntd.0006458.s005.docx]

**S2 Text - Table A.** Main characteristics of potentially relevant studies (n=6) reporting efficacy data against STH infections from ivermectin-albendazole co-administration

| **Publication** | **Country** | **Study type** | **Treated parasite** | **Age/Study population** | **Treatment regimen** | **Follow-up period** | **ALB or IVM alone comparator** | **Stool sampling (diagnostics)** | **Participants and efficacy data (No. treated/CR/ERR)** | **Inclusion/Exclusion** |
| --- | --- | --- | --- | --- | --- | --- | --- | --- | --- | --- |
| Beach *et al.* 1999 | Haiti | RCT | asc, hk, tri | 5-11 years | IVM: 200-400µg/kg;  ALB: 400mg | 5 weeks | yes/yes | 1 at baseline – 1 at follow-up (mod. Stoll) | asc: n=73, CR=100%, ERR=100%  hk: n=17, CR=100%, ERR=100%  tri: n=93, CR=79.6%, ERR=68.0% | Excluded – not recommended dose |
| Belizario *et al.* 2003 | Philippines | RCT | asc, tri | 6-12 years | IVM: 200µg/kg;  ALB: 400mg | 7-14 days | yes/yes | 1 at baseline – 1 at follow-up (Kato-Katz) | asc: n=105, CR=78.1%, ERR=99.5%  tri: n=149, CR=65.1%, ERR=97.5% | Included in meta-analysis |
| Ismail *et al.* 1999 | Sri Lanka | RCT | tri | 4-14 years | IVM: 200µg/kg;  ALB: 400mg | 3 weeks | yes/no | 1 at baseline – 1 at follow-up (Kato-Katz) | tri: n=53, CR=79.3%, ERR=93.8% | Included in meta-analysis |
| Knopp *et al.* 2010 | Tanzania | RCT | asc, hk, tri | 5-16 years | IVM: 200µg/kg;  ALB: 400mg | 3 weeks | yes/no | 2 at baseline – 2 at follow-up (Kato-Katz) | asc: n=14, CR=92.9%, ERR=99.9%  hk: n=30, CR=66.7%, ERR=96%  tri: n=140, CR=37.9%, ERR=91.1% | Included in meta-analysis |
| Ndyomµgyenyi *et al.* 2008 | Uganda | RCT | asc, hk, tri | 15-49 years (pregnant women) | IVM: height;  ALB: 400mg | 3 weeks | yes/yes | 1 at baseline – 1 at follow-up (Kato-Katz) | asc: n=0, CR=NA, ERR=NA  hk: n=188, CR=92.6%, ERR=NR  tri: n=17, CR=70.6%, ERR=NR | Excluded – not recommended dose |
| Speich *et al.* 2015 | Tanzania | RCT | asc, hk, tri | 6-14 years | IVM: 200µg/kg;  ALB: 400mg | 18-23 days | no/no | 2 at baseline – 2 at follow-up (Kato-Katz) | asc: n=50, CR=98%, ERR=100%  hk: n=42, CR=50%, ERR=95.4%  tri: n=109, CR=27.5%, ERR=94.5% | Included – efficacy parameters for combination therapy |

ALB: albendazole, asc: *Ascaris lumbricoides*, CR: cure rate, ERR: egg reduction rate, hk: hookworm, IVM: ivermectin, NA: not applicable; NR: not reported/detailed, RCT: randomized-controlled trial, tri: *Trichuris trichiura*

**S2 Text - Table B.** Main characteristics of potentially relevant studies (n=32) reporting safety data on ivermectin-albendazole co-administration

| **Publication** | **Country** | **Study type** | **Treated parasite** | **Age/Study population** | **Treatment regimen** | **Time span (follow-up post-treatment)** | **ALB or IVM alone comparator** | **Surveillance method** | **Participants and AE data (No. treated/No. AEs/No. SAEs)** | **Inclusion/Exclusion** |
| --- | --- | --- | --- | --- | --- | --- | --- | --- | --- | --- |
| Addiss *et al.* 1997 | Haiti | RCT | wb | 5-11 years | IVM: 200-400µg/kg;  ALB: 400mg | every day during 3-5 days (high mf patients: every 4-6h | yes/yes | active | lf-positive children 44/NR/NR | Included – Qualitative appraisal/Frequencies of symptoms |
| Amsden *et al.* 2007 | USA | randomized, open-label, three-way crossover trial | - | ≥18 years | IVM: 200-400µg/kg;  ALB: 400mg | 7 days | no/no | active | healthy adult subjects (lf-non endemic area)  18/1/0 | Included – Qualitative appraisal/Frequencies of symptoms |
| Anto *et al.* 2011 | Ghana | Clinical trial with matched groups | sh, sm, (wb, onc) | ≥ 5 years | IVM: height;  ALB: MDA standard dose | 2 weeks (+ 3 months) | no/no | passive | community members 15552/130/0 | Included – Qualitative appraisal/Frequencies of symptoms |
| Asio *et al.* 2009a | Uganda | Clinical trial with matched groups | mp | 9-77 years | IVM: 150-200µg/kg;  ALB: 400mg | 7 days (day 0, 1, 3, and 6) | yes/yes | active | mp-infected individuals 15/0/0 | Included – Qualitative appraisal/Frequencies of symptoms (excluded from meta-analysis due to zero AEs in all groups) |
| Asio *et al.* 2009b | Uganda | RCT | mp | 5-77 years | IVM: 150-200µg/kg;  ALB: 400mg | 7 days | no/yes | passive | mp-infected community members 86/0/0 | Included – Qualitative appraisal (excluded from meta-analysis due to zero AEs in all groups) |
| Awadzi *et al.* 1995 | Ghana | RCT | onc | 15-62 years | IVM: 150µg/kg;  ALB: 800mg | 18 days | no/yes | active | onc-infected male patients  (NA) | Excluded – IVM + ALB not co-administered (ALB 1 week later) |
| Awadzi *et al.* 2003 | Ghana | RCT | onc | 22-54 years (men only) | IVM: 2x6mg tablets;  ALB: 400mg | Mazotti-reaction scores: 30days AEs: 9 days (day1, 2, 3 or 8) | yes/yes | active | onc-infected men 14/14/0 | Included – Quantitative (meta-analysis) and qualitative appraisal (frequencies of symptoms) |
| Belizario *et al.* 2003 | Philippines | RCT | sth | 6-12 years | IVM: 200µg/kg;  ALB: 400mg | 7-14 days | yes/yes | active | sth-infected children  151/NR/NR | Excluded – too much missing information, safety assessed but not reported in the article |
| Coulibaly *et al.* 2015 | Mali | prospective cross-sectional study | wb | ≥ 5 years | IVM: MDA standard dose;  ALB: MDA standard dose | NR | no/no | NR | population at risk of lf-infection  2135/13/0 | Included – Qualitative appraisal |
| Dembele *et al.* 2010 | Mali | RCT | wb, mp | 18-62 years | 1) IVM: 150µg/kg;  ALB: 400mg  2) IVM: 400µg/kg;  ALB: 800mg | 7 days | no/no | active | lf-infected adults  42/9/0 | Included – Qualitative appraisal/Frequencies of symptoms |
| Dunyo *et al.* 2000 | Ghana | RCT | wb | 6-84 years | IVM: 150-200µg/kg;  ALB: 400mg | starting at 12h for the next 5 days | yes/yes | active | lf-positive and lf-negative individuals 332/47/0 | Included – Quantitative (meta-analysis) and qualitative appraisal (frequencies of symptoms) |
| Gyapong *et al.* 2003 | Ghana | Retrospective observational trial (after MDA campaign) | wb | infants (0-42 weeks) | IVM: MDA standard dose;  ALB: MDA standard dose | 42 weeks | no/no | active | 343 pregnancies, whereof 50 inadvertently treated (40 followed live births/1 CongMal) | Included – Qualitative appraisal (additional outcomes) |
| Hodges *et al.* 2010 | Sierra Leone | Reporting after MDA campaign | wb | ≥ 5 years | IVM: MDA standard dose;  ALB: MDA standard dose | 5 days | no/no | passive and active | population at risk of lf infection (1104407/146/1) | Included – Qualitative appraisal/Frequencies of symptoms |
| Horton *et al.* 2000 | Ecuador, Gabon, Ghana, Haiti, India, Philippines, Sri Lanka, Tanzania | Review (various study types) | sth, lf, onc | various | IVM: various;  ALB: various | various | EC: yes/yes  GA: yes/yes  PH: yes/yes  LK: yes/no | passive and active | Not published elsewhere:  Ecuador (Espinel): 122/NR/NR  Gabon (Richard-Lenoble): 181/NR/NR  Philippines (Belizario): 155/NR/NR  Sri Lanka (Weerasooriya): 32/NR/NR | Included - Provides complementary data for Ismail *et al.* 1998 |
| Ismail *et al.* 1998 | Sri Lanka | Clinical trial (blinded) | wb | 18-58 years | IVM: 400µg/kg;  ALB: 600mg | 5 days (4x/day during first 48h) | yes/no | active | lf-positive men  (13/NR/0) | Included – Qualitative appraisal/Frequencies of symptoms |
| Ismail *et al.* 2001 | Sri Lanka | Clinical trial (blinded) | wb | 18-58 years | 1) IVM: 200µg/kg;  ALB: 400mg  2) IVM: 400µg/kg;  ALB: 600mg | symptoms: 3 days  clinical ex.: 2 weeks, 1, 2, 3, 6, 9, 12, 15, 18 and 24 months | no/no | active | lf-positive men   1. (16/NR/0) 2. (15/NR/0) | Included – Qualitative appraisal/Frequencies of symptoms |
| Keiser *et al.* 2003 | Mali | Clinical trial | wb, mp | 18-65 years | IVM: 200µg/kg;  ALB: 400mg | 5 days (2, 4, 6, 8, 12, 24, 36, 48, 72, 96 and 120h) | no/no | active | lf- or/and mp-positive and negative adults (40/11/0) | Included – Qualitative appraisal/Frequencies of symptoms |
| Knopp *et al.* 2010 | Tanzania | RCT | tri | 5-16 years | IVM: 200µg/kg;  ALB: 400mg | 48h | yes/no | active | *Trichuris*-infected schoolchildren (144/64/0) | Included – Quantitative (meta-analysis) and qualitative appraisal (frequencies of symptoms) |
| Makunde *et al.* 2003 | Tanzania | RCT | wb, onc | 15-55 years | IVM: 150µg/kg;  ALB: 400mg | every 6h during 48h | yes/no | active | lf-single vs. lf/onc-co-infected individuals (20/11/0) | Included – Quantitative (meta-analysis) and qualitative appraisal (frequencies of symptoms) |
| Na-Bangchang *et al.* 2006 | Thailand | Clinical trial (open, randomised) | - | 18-55 years | IVM: 200µg/kg;  ALB: 400mg | 8 days | no/no | active | healthy subjects (23/0/0) | Included – Qualitative appraisal/Frequencies of symptoms |
| Namwanje *et al.* 2011 | Uganda | RCT | sm, sth, wb | 5-18 years | IVM: 200µg/kg;  ALB: 400mg | 7 days (daily) | no/no | active | children infected with lf alone  (22/NR/0) | Excluded - missing/non-extractable data (data not distinguishable with group who received supplemental drug) |
| Ndyomµgyenyi *et al.* 2008 | Uganda | RCT | sth | 15-49 years (women of childbearing age) | IVM: height;  ALB: 400mg | first exam: at ≥16 weeks gestational age, last exam: 1 month after birth | yes/yes | passive | sth-infected pregnant women  (199/8/0) | Included – Quantitative (meta-analysis) and qualitative appraisal (frequencies of symptoms, additional outcomes) |
| Rodriguez-Guardado *et al.* 2012 | Spain | hospital-based prospective observational study | ss | NR | IVM: 200µg/kg;  ALB: 400mg (repeated treatments) | 2 weeks, 1, 3 , 6, 9 and 12 months | no/no | active | ss-infected patients (81/NR/0) | Excluded - missing/non-extractable data (abstract of poster session only) |
| Shenoy *et al.* 1999 | India | Clinical trial (open, hospital-based) | bm | 14-70 years | IVM: 200µg/kg;  ALB: 400mg | 7 days | (yes, only n=3 in this group!) /no | active | lf-positive individuals (16/12/0) | Included – Qualitative appraisal/Frequencies of symptoms |
| Shenoy *et al.* 2000 | India | Clinical trial (open, hospital-based) | bm | 14-70 years | IVM: 200µg/kg;  ALB: 400mg | 5 days | no/no | active | lf-positive individuals (12/6/0) | Included – Qualitative appraisal/Frequencies of symptoms |
| Simonsen *et al.* 2004 | Tanzania | RCT | wb | 6-18 years | IVM: 150-200µg/kg;  ALB: 400mg | every day during 5 days | no/yes | passive | lf-positive and lf-negative pupils (586/NR/0) | Included – Qualitative appraisal/Frequencies of symptoms |
| Speich *et al.* 2015 | Tanzania | RCT | tri | 6-14 years | IVM: 200µg/kg;  ALB: 400mg | 3h and 24h | no/no | active | Trichuris-infected pupils (108/22/0) | Included – Qualitative appraisal/Frequencies of symptoms |
| Tafatatha *et al.* 2015 | Malawi | RCT | wb | 18-55 years | IVM: 200-400µg/kg;  ALB: 400-800mg | 7 days | no/no | passive and active | lf-positive adults (70/22/0) | Included – Qualitative appraisal/Frequencies of symptoms |
| Thomsen *et al.* 2016 | Papua New Guinea | RCT | wb | 18-60 years | IVM: 200µg/kg;  ALB: 400mg  +DEC: 6mg/kg | 7 days (4, 8, 12, 24, 48, 72, 168h) | no/no | active | lf-positive adults  (NA) | Excluded – Supplementary drug (DEC) in combination group |
| Turner *et al.* 2006 | Ghana | RCT | wb, wbb | 18-70 years | IVM: 150µg/kg;  ALB: 400mg | 48h | no/no | active | lf-positive adults (28/20/0) | Included – Qualitative appraisal/Frequencies of symptoms |
| Wen *et al.* 2008 | China | RCT | sth | 6-70 years | IVM: 100-200 µg/kg;  ALB: 400mg | 24h | yes/yes | active | STH-positive farmers and children  (NA) | Excluded - IVM + ALB not co-administered |
| WHO *et al.* 2003 | Burkina Faso, Nigeria, Tanzania | Post-treatment report from national control programs | lf | ≥ 5 years | IVM: MDA standard dose;  ALB: MDA standard dose | NR | no/no | active | population at risk of lf (9831/2358/NR) | Included – Qualitative appraisal/Frequencies of symptoms |

AE: adverse event, ALB: Albendazole, IVM: Ivermectin, MDA: mass drug administration, NR: Not reported/detailed, RCT: randomized controlled trial, SAE: serious adverse event. Parasitic diseases: bm=*Brugia malayi*, lf=lymphatic filariasis (species not specified), mp=*Mansonella perstans*, onc=*Onchorcerca volvulus*, sh=*Schistosoma haematobium*, sm=*Schistosoma mansoni*, ss=*Strongyloides stercoralis*, sth=soil-transmitted helminths, tri=*Trichuris*, wb=*Wuchereria bancrofti,* wbb=*Wolbachia* bacteria

**Alphabetized list of full citations for papers included in S1 Tables A & B**

1. Addiss DG, Beach MJ, Streit TG, Lutwick S, LeConte FH, Lafontant JG, et al. Randomised placebo-controlled comparison of ivermectin and albendazole alone and in combination for Wuchereria bancrofti microfilaraemia in Haitian children. Lancet. 1997;350(9076):480-4. doi: 10.1016/s0140-6736(97)02231-9. PubMed PMID: 9274584.

2. Amsden GW, Gregory TB, Michalak CA, Glue P, Knirsch CA. Pharmacokinetics of azithromycin and the combination of ivermectin and albendazole when administered alone and concurrently in healthy volunteers. Am J Trop Med Hyg. 2007;76(6):1153-7. PubMed PMID: 17556628.

3. Anto F, Asoala V, Anyorigiya T, Oduro A, Adjuik M, Akweongo P, et al. Simultaneous administration of praziquantel, ivermectin and albendazole, in a community in rural northern Ghana endemic for schistosomiasis, onchocerciasis and lymphatic filariasis. Trop Med Int Health. 2011;16(9):1112-9. doi: 10.1111/j.1365-3156.2011.02814.x. PubMed PMID: 21689221.

4. Asio SM, Simonsen PE, Onapa AW. Mansonella perstans: safety and efficacy of ivermectin alone, albendazole alone and the two drugs in combination. Ann Trop Med Parasitol. 2009;103(1):31-7. doi: 10.1179/136485909x384929. PubMed PMID: 19173774.

5. Asio SM, Simonsen PE, Onapa AW. A randomised, double-blind field trial of ivermectin alone and in combination with albendazole for the treatment of Mansonella perstans infections in Uganda. Trans R Soc Trop Med Hyg. 2009;103(3):274-9. doi: 10.1016/j.trstmh.2008.10.038. PubMed PMID: 19081121.

6. Awadzi K, Opoku NO, Addy ET, Quartey BT. The chemotherapy of onchocerciasis. XIX: The clinical and laboratory tolerance of high dose ivermectin. Trop Med Parasitol. 1995;46(2):131-7. PubMed PMID: 8525285.

7. Awadzi K, Edwards G, Duke BO, Opoku NO, Attah SK, Addy ET, et al. The co-administration of ivermectin and albendazole--safety, pharmacokinetics and efficacy against Onchocerca volvulus. Ann Trop Med Parasitol. 2003;97(2):165-78. doi: 10.1179/000349803235001697. PubMed PMID: 12803872.

8. Beach MJ, Streit TG, Addiss DG, Prospere R, Roberts JM, Lammie PJ. Assessment of combined ivermectin and albendazole for treatment of intestinal helminth and Wuchereria bancrofti infections in Haitian schoolchildren. Am J Trop Med Hyg. 1999;60(3):479-86. PubMed PMID: 10466981.

9. Belizario VY, Amarillo ME, de Leon WU, de los Reyes AE, Bugayong MG, Macatangay BJ. A comparison of the efficacy of single doses of albendazole, ivermectin, and diethylcarbamazine alone or in combinations against Ascaris and Trichuris spp. Bull World Health Organ. 2003;81(1):35-42. PubMed PMID: 12640474.

10. Coulibaly YI, Dembele B, Diallo AA, Konate S, Dolo H, Coulibaly SY, et al. The Impact of Six Annual Rounds of Mass Drug Administration on Wuchereria bancrofti Infections in Humans and in Mosquitoes in Mali. Am J Trop Med Hyg. 2015;93(2):356-60. doi: 10.4269/ajtmh.14-0516. PubMed PMID: 26033027.

11. Dembele B, Coulibaly YI, Dolo H, Konate S, Coulibaly SY, Sanogo D, et al. Use of high-dose, twice-yearly albendazole and ivermectin to suppress Wuchereria bancrofti microfilarial levels. Clin Infect Dis. 2010;51(11):1229-35. doi: 10.1086/657063. PubMed PMID: 21039220.

12. Dunyo SK, Nkrumah FK, Simonsen PE. A randomized double-blind placebo-controlled field trial of ivermectin and albendazole alone and in combination for the treatment of lymphatic filariasis in Ghana. Trans R Soc Trop Med Hyg. 2000;94(2):205-11. PubMed PMID: 10897370.

13. Gyapong JO, Chinbuah MA, Gyapong M. Inadvertent exposure of pregnant women to ivermectin and albendazole during mass drug administration for lymphatic filariasis. Trop Med Int Health. 2003;8(12):1093-101. PubMed PMID: 14641844.

14. Hodges MH, Smith SJ, Fussum D, Koroma JB, Conteh A, Sonnie M, et al. High coverage of mass drug administration for lymphatic filariasis in rural and non-rural settings in the Western Area, Sierra Leone. Parasit Vectors. 2010;3:120. doi: 10.1186/1756-3305-3-120. PubMed PMID: 21162751.

15. Horton J, Witt C, Ottesen EA, Lazdins JK, Addiss DG, Awadzi K, et al. An analysis of the safety of the single dose, two drug regimens used in programmes to eliminate lymphatic filariasis. Parasitology. 2000;121 Suppl:S147-60. PubMed PMID: 11386686.

16. Ismail MM, Jayakody RL, Weil GJ, Nirmalan N, Jayasinghe KS, Abeyewickrema W, et al. Efficacy of single dose combinations of albendazole, ivermectin and diethylcarbamazine for the treatment of bancroftian filariasis. Trans R Soc Trop Med Hyg. 1998;92(1):94-7. PubMed PMID: 9692166.

17. Ismail MM, Jayakody RL. Efficacy of albendazole and its combinations with ivermectin or diethylcarbamazine (DEC) in the treatment of Trichuris trichiura infections in Sri Lanka. Ann Trop Med Parasitol. 1999;93(5):501-4. PubMed PMID: 10690245.

18. Ismail MM, Jayakody RL, Weil GJ, Fernando D, De Silva MSG, De Silva GAC, et al. Long-term efficacy of single-dose combinations of albendazole, ivermectin and diethylcarbamazine for the treatment of bancroftian filariasis. Transactions of the Royal Society of Tropical Medicine and Hygiene. 2001;95(3):332-5. doi: https://doi.org/10.1016/S0035-9203(01)90257-3.

19. Keiser PB, Coulibaly YI, Keita F, Traore D, Diallo A, Diallo DA, et al. Clinical characteristics of post-treatment reactions to ivermectin/albendazole for Wuchereria bancrofti in a region co-endemic for Mansonella perstans. Am J Trop Med Hyg. 2003;69(3):331-5. PubMed PMID: 14628953.

20. Knopp S, Mohammed KA, Speich B, Hattendorf J, Khamis IS, Khamis AN, et al. Albendazole and mebendazole administered alone or in combination with ivermectin against Trichuris trichiura: a randomized controlled trial. Clin Infect Dis. 2010;51(12):1420-8. doi: 10.1086/657310. PubMed PMID: 21062129.

21. Makunde WH, Kamugisha LM, Massaga JJ, Makunde RW, Savael ZX, Akida J, et al. Treatment of co-infection with bancroftian filariasis and onchocerciasis: a safety and efficacy study of albendazole with ivermectin compared to treatment of single infection with bancroftian filariasis. Filaria J. 2003;2(1):15. doi: 10.1186/1475-2883-2-15. PubMed PMID: 14613509.

22. Na-Bangchang K, Kietinun S, Pawa KK, Hanpitakpong W, Na-Bangchang C, Lazdins J. Assessments of pharmacokinetic drug interactions and tolerability of albendazole, praziquantel and ivermectin combinations. Trans R Soc Trop Med Hyg. 2006;100(4):335-45. doi: 10.1016/j.trstmh.2005.05.017. PubMed PMID: 16271272.

23. Namwanje H, Kabatereine N, Olsen A. A randomised controlled clinical trial on the safety of co-administration of albendazole, ivermectin and praziquantel in infected schoolchildren in Uganda. Trans R Soc Trop Med Hyg. 2011;105(4):181-8. doi: 10.1016/j.trstmh.2010.11.012. PubMed PMID: 21353271.

24. Ndyomugyenyi R, Kabatereine N, Olsen A, Magnussen P. Efficacy of ivermectin and albendazole alone and in combination for treatment of soil-transmitted helminths in pregnancy and adverse events: a randomized open label controlled intervention trial in Masindi district, western Uganda. Am J Trop Med Hyg. 2008;79(6):856-63. PubMed PMID: 19052293.

25. Rodriguez-Guardado A, Rodriguez M, Pérez F, Martinez M, Morán N, Carcaba V, et al. Efficacy and safety of combined therapy with albendazol and ivermectin in chronic strongyloidiasis: observational study. Clinical Microbiology and Infection. 2012;18, Supplement 3:114-715. doi: https://doi.org/10.1111/j.1469-0691.2012.03802.x.

26. Rowbotton ARA, Verde O, Ache LJ, Gonzalez J, Guerra A, Sanchez V, et al. Evaluation of the efficacy of antihelmintic drugs for the control of Trichuris trichiura and other helminthic diseases in the state of Aragua, Venezuela. Boletin De Malariologia Y Salud Ambiental. 2012;52(2):195-209. PubMed PMID: WOS:000328161000002.

27. Shenoy RK, Dalia S, John A, Suma TK, Kumaraswami V. Treatment of the microfilaraemia of asymptomatic brugian filariasis with single doses of ivermectin, diethylcarbamazine or albendazole, in various combinations. Ann Trop Med Parasitol. 1999;93(6):643-51. PubMed PMID: 10707109.

28. Shenoy RK, John A, Babu BS, Suma TK, Kumaraswami V. Two-year follow-up of the microfilaraemia of asymptomatic brugian filariasis, after treatment with two, annual, single doses of ivermectin, diethylcarbamazine and albendazole, in various combinations. Ann Trop Med Parasitol. 2000;94(6):607-14. PubMed PMID: 11064762.

29. Simonsen PE, Magesa SM, Dunyo SK, Malecela-Lazaro MN, Michael E. The effect of single dose ivermectin alone or in combination with albendazole on Wuchereria bancrofti infection in primary school children in Tanzania. Trans R Soc Trop Med Hyg. 2004;98(8):462-72. doi: 10.1016/j.trstmh.2003.12.005. PubMed PMID: 15186934.

30. Speich B, Ali SM, Ame SM, Bogoch, II, Alles R, Huwyler J, et al. Efficacy and safety of albendazole plus ivermectin, albendazole plus mebendazole, albendazole plus oxantel pamoate, and mebendazole alone against *Trichuris trichiura* and concomitant soil-transmitted helminth infections: a four-arm, randomised controlled trial. Lancet Infect Dis. 2015;15(3):277-84. doi: 10.1016/s1473-3099(14)71050-3. PubMed PMID: 25589326.

31. Speich B, Moser W, Ali SM, Ame SM, Albonico M, Hattendorf J, et al. Efficacy and reinfection with soil-transmitted helminths 18-weeks post-treatment with albendazole-ivermectin, albendazole-mebendazole, albendazole-oxantel pamoate and mebendazole. Parasit Vectors. 2016;9:123. doi: 10.1186/s13071-016-1406-8. PubMed PMID: 26935065.

32. Tafatatha TT, Ngwira BM, Taegtmeyer M, Phiri AJ, Wilson TP, Banda LG, et al. Randomised controlled clinical trial of increased dose and frequency of albendazole and ivermectin on Wuchereria bancrofti microfilarial clearance in northern Malawi. Trans R Soc Trop Med Hyg. 2015;109(6):393-9. doi: 10.1093/trstmh/trv027. PubMed PMID: 25877874.

33. Thomsen EK, Sanuku N, Baea M, Satofan S, Maki E, Lombore B, et al. Efficacy, Safety, and Pharmacokinetics of Coadministered Diethylcarbamazine, Albendazole, and Ivermectin for Treatment of Bancroftian Filariasis. Clin Infect Dis. 2016;62(3):334-41. doi: 10.1093/cid/civ882. PubMed PMID: 26486704.

34. Turner JD, Mand S, Debrah AY, Muehlfeld J, Pfarr K, McGarry HF, et al. A randomized, double-blind clinical trial of a 3-week course of doxycycline plus albendazole and ivermectin for the treatment of Wuchereria bancrofti infection. Clin Infect Dis. 2006;42(8):1081-9. doi: 10.1086/501351. PubMed PMID: 16575724.

35. Wen LY, Yan XL, Sun FH, Fang YY, Yang MJ, Lou LJ. A randomized, double-blind, multicenter clinical trial on the efficacy of ivermectin against intestinal nematode infections in China. Acta Trop. 2008;106(3):190-4. doi: 10.1016/j.actatropica.2008.03.007. PubMed PMID: 18452885.

36. WHO. Report on active surveillance for adverse events following the use of drug co-administrations in the global programme to eliminate lymphatic filariasis. Wkly Epidemiol Rec. 2003;78(36):315-7. PubMed PMID: 14518107.
